# Clonal CD8 T cells link peripheral and intrathecal immunity in ALS4 progression: a multiomics reference for ALS

**DOI:** 10.21203/rs.3.rs-10810896/v1

**Published:** 2026-09-09

**Authors:** Marina Terekhova, Rima Melhem, Carisa Zeng, Jessie Martin, Tanya Lizbeth Joseph, Pavla Bohacova, Melina Jones, Avindra Nath, Nicole Benoit, George Harmison, Gregory F. Wu, Alexey Sergushichev, Maxim N. Artyomov, Christopher Grunseich, Laura Campisi

**Affiliations:** 1 Department of Pathology and Immunology, Washington University in Saint Louis, Saint Louis, Missouri, USA; 2 Department of Developmental Biology, Washington University in Saint Louis, Saint Louis, Missouri, USA (present address); 3 Medical Oncology, Washington University School of Medicine in Saint Louis, Saint Louis, Missouri, USA (present address); 4 Section of Infections of The Nervous System, National Institute of Neurological Disorders and Stroke, National Institutes of Health, Bethesda, Maryland, USA; 5 Biospecimen Core, National Institutes of Neurological Disorders and Stroke, National Institutes of Health, Bethesda, Maryland, USA; 6 Neurogenetics Branch, National Institute of Neurological Disorders and Stroke, National Institutes of Health, Bethesda, Maryland, US; 7 Department of Neurology, Washington University School of Medicine, St. Louis, Missouri, USA; 8 Neurology Service, Veterans Affairs St. Louis Health Care System, St. Louis, Missouri, USA; 9 Brain Immunology & Glia Center, Washington University in St. Louis, St, Louis, Missouri, USA; 10 Hope Center for Neurological Disorders, Washington University in St. Louis, St, Louis, Missouri, USA; 11 ALS Center, Washington University in St. Louis, St, Louis, Missouri, USA

## Abstract

Dysregulated immune responses increasingly appear central to amyotrophic lateral sclerosis (ALS) pathology, but rapid progression and delayed diagnosis limit our understanding of how immune events evolve over the course of disease. By combining high-parameter spectral flow cytometry with single-cell RNA, TCR and BCR sequencing of peripheral blood and cerebrospinal fluid (CSF), we map the immune landscape in ALS4, a juvenile-onset, slowly progressive ALS subtype, providing the first view of immune states spanning decades of disease progression. We identify an early-emerging, progressively amplifying CD8 T cell program characterized by clonal expansion of peripheral GZMK^+^ and GZMB^+^ subsets and an abnormally high degree of TCR clonotype sharing between GZMK^+^ CSF and GZMB^+^ blood CD8 T cells. Our work defines a CD8 T cell trajectory that parallels clinical progression in ALS4 across CSF and blood and provides a reference for comparison with other ALS subtypes.

## Introduction

Amyotrophic lateral sclerosis (ALS) is a heterogeneous neurodegenerative disease characterized by degeneration of upper and lower motor neurons, progressive muscle atrophy, and death from respiratory failure^1–3^. ALS is predominantly sporadic, with >90% of cases lacking a clear familial history. Although over 40 genes have been associated with different forms of the disease, a single high-penetrance genetic cause is not identified in most patients^1–3^. Median survival in ALS is 3-5 years from symptom onset and incidence is ~2-3 per 100,000 individuals, with case numbers projected to rise substantially over the coming decades^4^. Despite intense effort, there are no definitive disease-modifying therapies, in part because the etiologic and pathogenic mechanisms underlying ALS remain incompletely defined. Beyond neuronal loss and glial activation, converging evidence implicates the peripheral immune system in ALS onset, progression, and spread^5–8^. Yet the timing, phenotype, and tissue distribution of adaptive immune responses in human ALS-and whether they act as biomarkers, drivers of neurodegeneration, or bystanders-remain unclear. In contrast to most ALS forms, in which late diagnosis and rapid clinical decline preclude systematic observation of early immune events in patients, ALS4 provides a uniquely tractable setting in which to dissect these mechanisms. ALS4 is a rare, juvenile-onset, autosomal-dominant, and highly penetrant motor neuron disease caused by pathogenic variants in the RNA-DNA helicase senataxin (encoded by *SETX*)^9–11^. ALS4 differs from “classical” ALS in its slow progression and preserved life expectancy, but affected individuals exhibit hallmark ALS features, including severe distal muscle weakness and atrophy with degeneration of both upper and lower motor neurons^9,11^. Because ALS4 is familial, juvenile, and slowly progressive, it affords a rare opportunity to study ongoing pathology across disease stages in living patients^11^. Moreover, *SETX* is well conserved between humans and mice, and knock-in mice expressing the ALS4-causative L389S mutation faithfully recapitulate key clinical and pathological aspects of the human disease^9,12^, establishing a robust platform for mechanistic and translational studies.

Using this ALS4 mouse model, we previously identified a disease-associated CD8 T cell signature characterized by clonally expanded, terminally differentiated effector memory CD8 T cells that track with motor neuron degeneration^12^. Consistent with these findings, we demonstrated that ALS4 patients exhibit an increased frequency of clonally expanded terminally differentiated CD8 T cells in peripheral blood at late disease stages^12^. Notably, other groups have independently reported T cell signatures in sporadic and familial ALS^13–21^, suggesting that immune alterations identified in ALS4 may generalize to broader ALS subtypes. Together, these observations raise fundamental questions: when do immune abnormalities first arise in human ALS, what are the dominant dysfunctional T cell subsets, and to what extent do peripheral immune signatures mirror immune processes within the central nervous system (CNS)?

To address these questions, we exploited the slow progression and early detectability of ALS4 to profile the immune landscape of patients in peripheral blood and cerebrospinal fluid (CSF) across disease stages in a unique cohort spanning 8-74 years of age, with age-matched healthy controls. We identified a CD8 T cell signature marked by pronounced clonal expansion and granzyme B (GZMB) expression in the periphery that tracks with clinical progression and is already detectable in childhood, consistent with an early and persistent antigen-driven response. Strikingly, we observed substantial overlap in CD8 T cell clonotypes between peripheral blood and CSF in ALS4 patients, but not in healthy controls. The highest degree of sharing was observed for CSF GZMK^+^ Tem cells, which mainly shared clonotypes with GZMB^+^ Tem cells in the blood, suggesting that a common pool of antigen-experienced CD8 T cells circulates between the two compartments. Furthermore, transcriptomic and clonotype analyses suggest that peripheral clonal expanded GZMK^+^ CD8 T cells gain features of terminally cytotoxic GZMB^+^ cells. In contrast, peripheral CD4 T cells exhibit a shift toward Th1/Th17 polarization without comparable clonal expansion or a prominent CSF counterpart, suggesting a qualitatively different pattern of immune remodeling for CD4 versus CD8 T cells in ALS4 pathophysiology.

This study is, to our knowledge, the first to delineate the evolution of the immune system over decades in any form of ALS and to integrate spectral flow cytometry and paired high-throughput TCR and transcriptomic profiling of peripheral blood and CSF from the same patients. By demonstrating that clonally expanded CD8 T cells constitute a major inflammatory correlate of ALS4 progression, our work establishes CD8 T cells as a tractable biomarker and candidate effector population for therapeutic targeting in ALS and may provide a framework for extending these insights to other ALS subtypes.

## Results

### Cohort stratification and study design.

We profiled immune changes across the full clinical spectrum of ALS4 in a deeply characterized cohort spanning 8-74 years of age (Fig. 1a, **Supplementary Table 1**). All ALS4 patients had weakness on neurological examination and exhibited features of disease consistent with a previously reported cross sectional analysis^11^. Peripheral blood was collected in parallel with standardized neurological and clinical assessments, and donors showed no evidence of active infection or relevant systemic comorbidities (**Supplementary Table 1**). All affected participants belonged to a single extended kindred and carried the SETX Leu389Ser (L389S) mutation, except for one sporadic ALS4 case harboring a distinct substitution at codon 384 (E384K), which has not been previously reported. Control samples were largely unrelated to affected individuals (one familial control) and were obtained from the NIH, Washington University in St. Louis, commercial vendors or public dataset (**Supplementary Table 2**).

We established high-dimensional immunophenotypic maps of peripheral blood mononuclear cells (PBMCs) using two complementary spectral flow cytometry panels. A 28-parameter “general” panel was designed to capture major innate and adaptive leukocyte populations and was applied to PBMCs from 18 ALS4 donors and 21 age-matched healthy controls (HC). In parallel, a 34-parameter T cell focused panel was optimized^12,22^ to resolve CD4 and CD8 T cell differentiation and activation states and was applied to samples from 17 ALS4 donors and 25 HC (Fig. 1a and **Supplementary Table 3**).

To define transcriptional programs and clonal features associated with discrete immune cell states, we complemented our analyses with single-cell RNA sequencing (scRNA-seq) coupled with paired single-cell B cell receptor (scBCR) and T cell receptor (scTCR) sequencing. Five to six ALS4 donors per age group (n = 17 total) and four controls per group (n = 12 total) were profiled, yielding 128,610 high-quality single cells (Fig. 1a,b and Extended Data Fig. 1a). To increase power for age-associated comparisons and provide an external reference, we additionally integrated PBMC scRNA-seq data from six healthy adults (32-75 years old) from a published dataset into our analysis^23^, which resulted in a total of 160,587 cells (Fig. 1a,b and Extended Data Fig. 1a).

To resolve stage-related changes in peripheral immune composition, we stratified participants by age, which is a proxy for disease stage^11^, into Early (E, ≤30 years), Middle (M, 31–59 years), and Late (L, ≥60 years) groups (Fig. 1a). As initial symptoms typically emerge in childhood or adolescence and progress with advancing age, chronological age served as a surrogate marker of disease stage in our analyses.

Depending on sample availability, individual donors contributed material for scRNA-seq, spectral flow cytometry (general and/or T cell-focused panels), or both, as detailed in **Supplementary Table 2**. For most donors, all three modalities were available and temporally matched.

To determine whether peripheral immune alterations mirrored inflammatory processes within the central nervous system, we performed matched scRNA-seq and scTCR-seq on cerebrospinal spinal fluid (CSF) from four ALS4 patients (ages 42, 47, 59 and 70 years). These data were analyzed together with CSF and PBMC profiles from 7 HC (Fig. 1, **Supplementary table 2**), (Fig. 4 and Extended Data Fig. 10).

### ALS4 progression is accompanied by increasing proportions of peripheral CD8 T cells.

We first profiled peripheral immune cell subsets in ALS4 patients and age-matched healthy controls (HC). Single cell analysis (Fig. 1b,c and Extended Data Fig. 1) together with spectral flow cytometry (Extended Data Fig. 2) allowed us to resolve all major immune populations across groups.

Overall, the proportional distribution in PBMCs of innate and innate-like immune cells (myeloid cells, natural killer (NK) cells, basophils, TCR*γδ* cells and mucosal-associated invariant T (MAIT) cells), as well as B cells and CD4 T cells, did not differ significantly over disease progression when comparing ALS4 patients to age-matched HC (Fig. 1c; Extended Data Figs. 1c and Extended Data Fig. 2–3). NK cells, TCRγδ cells, and myeloid subsets were further subclustered but likewise showed no consistent disease-associated shifts in their relative frequencies (Extended Data Figs. 2b,c and Extended Data Fig. 3).

Regarding the B cell compartment, we observed a reduction in total B cell frequency only in middle-stage patients compared to controls (Fig. 1c, Extended Data Fig. 1c). Spectral cytometry and scRNA-seq identified naïve, memory, plasmablast/plasma cell, CD11c^+^/T-bet^+/−^, activated, atypical, and transitional B cells, without differences in subset abundance or clonal expansion with disease progression (Extended Data Fig. 4a–e). We noted a heat shock protein (HSP)^+^ B cell population with high heat shock protein expression that was almost exclusively present in patients and not clonally expanded (Extended Data Fig. 4c–e). Given that HSPs are induced by cellular stress and senataxin maintains genome integrity during DNA replication^24^, we interpret these HSP^+^ B cells as reflecting heightened SETX-driven stress under physiological or technical conditions (e.g. cryopreservation), rather than a disease-specific neurodegenerative signature.

Our profiles of innate immune cells, CD4 T and B cells align with findings from a recent study that examined peripheral immune composition in patients with sporadic ALS and those with C9orf72-associated familial ALS, in which the C9orf72 repeat expansion represents the most common genetic cause of familial ALS^19^. Notably, the same study uncovered ALS-associated transcriptional alterations, particularly within monocyte populations^19^. We therefore asked whether similar differentially expressed (DE) genes could be detected in our cohort. Because ALS4 samples were available only from the NIH, disease status was nested for batch-correction. To avoid compromising the biological signal through the batch correction, we restricted DE analysis to ALS4 and control samples from the NIH cohort processed in the first sequencing batch. Within this subset, conventional monocytes exhibited the greatest degree of transcriptional change relative to other immune populations (Extended Data Fig. 5b). The ALS4 conventional monocyte signature showed significant enrichment of previously reported CD14^+^ monocyte signatures from C9orf72-ALS cohorts^19^ (and vice versa), including upregulation of genes associated with TNF/NF-*κ*B signaling and inflammatory response pathways (Extended Data Fig. 5c–e). Given the limited sample size, these DE results suggest, but do not definitively establish, a shared monocyte program between ALS4 and C9orf72 ALS, and should be viewed as supportive and hypothesis-generating.

In contrast to the relative stability of other immune compartments, we observed a significant increase in the proportion of CD8 T cells in ALS4 patients (Fig. 1c). This change began as a mild elevation in the younger group and became statistically significant over the course of disease (Fig. 1c), suggesting that the underlying process is initiated early in ALS4 but only becomes readily detectable at intermediate disease stages. Therefore, these findings indicate that CD8 T cells represent a prominent immunological abnormality during ALS4 progression.

### Early cytotoxic skewing and terminal differentiation of CD8 T cells in ALS4.

To better characterize the phenotype of ALS4-associated peripheral CD8 T cells, we next built on our previous work demonstrating that clonally expanded CD45RA^high/+^CD28^−^ CD8 T cells with a terminal effector phenotype are a hallmark of ALS4 patients at late disease stages^12^, we next asked whether analogous perturbations are already detectable at earlier disease stages. Spectral flow cytometry in our longitudinal cohort revealed that aberrantly increased frequencies of CD45RA^high/+^CD28^−^ CD8 T cells are already present in PBMCs from patients at the early (E) stage and continue to rise with age relative to HC (Fig. 1d and Extended Data Fig. 6a). These data indicate that an aberrant CD8 T cell signature is established in ALS4 patients at young ages and amplifies with disease progression.

To accurately resolve CD8 T cell subset heterogeneity across ALS4 disease progression, we employed scRNA-seq (Fig. 2). A smaller cohort of patients and controls was also profiled using sorted CD8 T cells instead of total PBMCs, yielding comparable results (Extended Data Fig. 6b,c). Through this scRNA-seq approach, we identified thirteen distinct CD8 T cell populations^22^: naïve cells (*CCR7*^high^*LEF1*^high^), recent thymic emigrants (RTE, *SOX4*^+^), central memory T cells (Tcm, *CCR7*^dim^*LEF1*^dim^) either expressing or lacking *CCR4*, tissue-resident memory T cells (Trm), memory cells (Tmem, *CCR7*^−^*LEF1*^dim^ GZMK+) expressing *KLRC2*, NKT-like cells (*NCAM1*^+^), effector memory T cells (Tem, *CCR7*^−^*LEF1*^−^Eomes^+^) expressing granzyme B or K (GZMB^+^ or GZMK^+^), HLA-DR^+^ GZMK^+^ effector memory cells, and TEMRA (*CCR7*^−^*LEF1*^−^*Eomes*^+^*KLRF1*^+^) cells (Fig. 2a). We observed a reduction in naïve CD8 T cells in ALS4 patients compared with controls -a trend beginning at an early age-along with a decline in RTE CD8 T cells in the middle (M) and late (L) ALS4 groups (Fig. 2b and Extended Data Fig. 6d). Conversely, GZMB-expressing clusters such as Tem GZMB^+^, Temra, and NKT-like cells were more abundant in M and L patients, surpassing the age-related changes observed in HC (Fig. 2b and Extended Data Fig. 6d). An increase in GZMK^+^ CD8 T cells was also evident in ALS4 patients compared with controls (Fig. 2b and Extended Data Fig. 6d). This CD8 T cell signature was also observed in the patient carrying the E384K mutation (indicated by a black circle).

Re-examination of our flow cytometry data further showed that CD45RA^^+^/high^CD28^−^ CD8 T cells constitute a heterogeneous compartment encompassing NKp80^+^ Temra cells^22^, GZMB^+^ effector memory cells, and GZMK^+^ populations, underscoring the limitations of using CD45RA and CD28 alone to define CD8 T cell subsets due to dynamic regulation during activation (Extended Data Fig. 6e). Although the increase did not reach statistical significance, GZMB^+^ CD8 T cells also tended to be higher in the early ALS4 group. Notably, analysis of two siblings aged 8 and 10 years, one carrying the L389S ALS4 mutation and the other unaffected, revealed a marked elevation of CD28^−^ GZMB^+^ CD8 T cells in the ALS4-affected child (Extended Data Fig. 6f).

In summary, our analysis reveals that ALS4 patients exhibit a distinct CD8 T cell signature characterized by early activation and terminal differentiation, with progressive accumulation of these cytotoxic populations as disease advances, only partly explained by aging.

### Clonal expansion of CD8 T cells in ALS4.

Using parallel single-cell T cell receptor (TCR) sequencing (scTCR-seq), we found that peripheral CD8 T cells from ALS4 patients were markedly clonally expanded, with this enrichment already apparent at the early (E) disease stage (Fig. 2c,d, Extended Data Fig. 7a). In the E group, 9% of CD8 T cells in ALS4 patients were classified as medium (identical TCR shared across 5-20 cells) or large expanded clones (20 < x ≤ 100 cells), whereas only a small fraction of medium expanded clones (<3.6%) was detected in age-matched controls. In the middle (M) stage, hyperexpanded CD8 T cells-defined as sharing an identical TCR sequence across more than 100 cells-increased to approximately 26% in patients versus about 1.4% in controls, and in the late (L) stage they exceeded 31% in patients compared with roughly 8% in controls (Fig. 2c). Although age-associated clonal expansion of CD8 T cells was observed in controls from E to L stages, the magnitude and early onset of clonal expansion in ALS4 patients indicate that this phenomenon cannot be explained by aging alone. Analysis of TCR usage across CD8 T cell subsets revealed that clonal expansion was highest and most consistent in NKT-like, Tem GZMB^+^, and Temra CD8 T cells, followed by GZMK^+^ CD8 T cells (Fig. 2c–d).

To determine whether clonally expanded CD8 T cells in ALS4 exhibit a unique transcriptional signature we performed within-donor comparisons in ALS4 patients, comparing low-versus highly expanded CD8 T cell clones across defined subpopulations (Fig. 2e). The most consistent expansion-associated transcriptional changes were observed in Tem GZMK^+^, Tem GZMB^+^, and TEMRA cells. In Tem GZMK^+^ cells, highly expanded clones upregulated *NKG7*, *KLRD1* (CD94), *PRF1* (perforin), and *GZMH* (granzyme H), indicating acquisition of a terminal effector, granule-rich cytotoxic program compatible with differentiation toward GZMB^+^ effector cells. In Tem GZMB^+^ cells, highly expanded clones showed features of chronically stimulated terminal effector T cells, with reduced expression of co-stimulatory/activation-linked genes (*KLRB1*, *JUNB*) and increased expression of the inhibitory/checkpoint receptor *KLRG1*^25^, alongside *AOAH* upregulation, suggesting adaptation to a lipid- and danger signal-rich, chronically inflamed environment^26^. Consistent with sustained antigenic exposure, *FCRL5*^27^ was also increased in these expanded Tem GZMB^+^ cells. In Temra cells, low-expanded clones expressed *GNLY* (granulysin), whereas highly expanded Temra clones showed downregulation of *TNFRSF9* (4-1BB/CD137), consistent with a late, highly cytotoxic but less co-stimulated, terminal effector state (Fig. 2e). Together, these within-donor pseudobulk analyses demonstrate that clonal expansion in ALS4 is tightly coupled to the emergence of highly cytotoxic, terminally differentiated CD8 Tem and Temra populations shaped by chronic antigen stimulation rather than by aging alone.

Cytomegalovirus (CMV)- and Epstein-Barr virus (EBV)-specific CD8 T cells have been described among clonally expanded populations in peripheral blood, cerebrospinal fluid, and meninges of individuals with Alzheimer’s disease^28,29^, suggesting that latent or prior microbial infection can drive clonal CD8 T cell expansion in neurodegenerative settings. However, in our ALS4 cohort, serological testing did not reveal evidence of active CMV infection (Supplementary Table 1). We further queried public databases of known TCR-antigen pairs and determined the frequency of CD3β clonotypes with matches in the VDJdb resource (Extended Data Fig. 7b and **Supplementary Table 4**). We found no evidence for increased overall CDR3β overlap with microbial-specific VDJdb sequences in ALS4 patients compared with healthy controls; in fact, the proportion of CDR3β overlap with CMV- and EBV-specific TCR sequences was slightly higher in controls, consistent with the absence of active cytomegalovirus infection in ALS4 patients (**Supplementary Table 1**) and our previous report^12^.

Because of the striking CD8 T cell clonal expansion and the familial nature of ALS4, we next investigated whether a specific HLA haplotype was associated with the disease. To increase power, we analyzed our current ALS4 cohort together with our previously published ALS4 dataset^12^ and a published dataset of sporadic ALS patients^30^ (**Supplementary Table 4**). We found that the DR7.DQ2.2 haplotype was present in approximately 50% of ALS4 patients, whereas it was not detected in sporadic ALS, indicating a potential ALS4-specific enrichment of this HLA background (Extended Data Fig. 7c and **Supplementary Table 5**). Interestingly, the patient carrying the E384K mutation also harbors the DR7.DQ2.2 haplotype. These data suggest that DR7.DQ2.2 may contribute to ALS4 risk or to its distinct immunologic signature, although it is not fully penetrant and therefore cannot by itself account for the ALS4 immune phenotype.

In conclusion, ALS4 progression is characterized by progressive clonal expansion of cytotoxic, chronically stimulated CD8 T cells, with no evidence for a dominant microbial-derived antigen.

### Peripheral Th1 and Th1/Th17 CD4 T cell skewing in ALS4.

We next profiled peripheral CD4 T cell subsets by spectral flow cytometry. Conventional CD4 T cells (CD25^−^) and regulatory T cells (Tregs; CD127^−^CD25^+^) were identified and each was subdivided into naïve (Fas^−^CD45RA^+^) and memory (Fas^+^) populations (Extended Data Fig. 8a and^22^). Conventional memory CD4 T cells were further partitioned into Temra (GZMB^+^), terminal effector GZMK^+^ cells, and functional helper subsets: Tfh (CXCR5^+^), Th22/Th17 (CCR10^+^), Th17 (CCR6^+^CXCR3^−^), Th1 (CCR6^−^CXCR3^+^), Th1/Th17 (CCR6^+^CXCR3^+^), and Th2 (CCR4^+^) cells (Extended Data Fig. 8a and^22^). Our analysis revealed a skewing toward Th1 and Th1/Th17 cells in ALS4 patients in middle and late groups as compared with HC, with a reduction in Th17 cells only in the M group (Fig. 3a and Extended Data Fig. 8b). These observations partially recapitulate earlier work in sporadic ALS reporting a peripheral Th1/Th17 bias^15^, but differ from a recent study describing reduced Th1 and Th1/Th17 cells in sporadic ALS^31^, suggesting that this CD4 T cell profile may be distinctive for ALS4.

To gain transcriptional resolution, we analyzed our scRNA-seq dataset, which identified 16 discrete CD4 T cell clusters (Fig. 3b). Unlike CD8 T cells, the relative abundance of naïve and recent thymic emigrant CD4 T cells did not differ between ALS4 patients and age-matched healthy controls (Fig. 3c and Extended Data Fig. 9). Consistent with flow cytometry, scRNA-seq showed increased representation of Th1 and Th1/Th17 clusters, but also a reduction in proliferative CD4 T cells at middle and late disease stages (Fig. 3d and Extended Data Fig. 9).

Prior studies in sporadic ALS have described clonal expansion of CD4 T cells^14^ and CD4 T cell recognition of C9orf72-derived antigens in peripheral blood^31^. We therefore analyzed paired scTCR-seq to assess whether the CD4 T cell changes in ALS4 reflected antigen-driven clonal expansion (Fig. 3e,f). As expected, CD4 T cell clonality increased with age in both groups and was largely restricted to terminal effector CD4 T cell clusters. Overall, clonality was comparable in both healthy controls and ALS4 patients (Fig. 3e,f), suggesting the absence of an overt CD4 T cell expansion in ALS4. However, analysis of clonotype sharing in peripheral blood revealed that a larger fraction of the terminal effector repertoire was shared with Th1/Th17 cells in ALS4 (14.8%) than in healthy controls (5.4%). This might suggest that terminal effector cells preferentially arise from Th1/Th17-associated clonotypes in ALS4.

Taken together, these data show that ALS4 progression is accompanied by a shift from homeostatic toward a pro-inflammatory CD4 T cell landscape, characterized by a skewing toward Th1 and Th1/Th17 subsets and loss of proliferative CD4 T cells, but not by overt antigen-driven clonal expansion of CD4 T cells.

### CD8 T cell repertoire sharing between CSF and blood.

Our previous analysis of postmortem tissue from two ALS4 patients -the only ALS4 postmortem samples currently available- revealed CD8 T cell infiltration in the ventral horn of the lumbar spinal cord, whereas CD8 T cells were absent from control tissues, including cortical brain regions from the same patients and spinal cords from non-ALS controls^12^. These observations, together with data from the ALS4 mouse model, indicated a CD8 T cell-associated immune signature spanning both the peripheral and central nervous systems of ALS4 patients^12^ and provided the rationale for a deeper characterization of these cells. To define the nature of CNS CD8 T cells and to determine whether immune perturbations in the CSF are mirrored in the peripheral immune compartment, we performed paired scRNA- and scTCR-seq on CSF (Fig. 1a, **Supplementary Table 2**). Because recruitment of healthy CSF donors is challenging, our dataset was jointly analyzed with a published reference cohort comprising paired blood and CSF immune cell transcriptomes from healthy individuals^23^, as already discussed above (Fig. 1a). In our cohort, 4,849 cells passed quality control and were integrated with the published reference, yielding a combined dataset of 24,797 cells (Fig. 4a, Extended Data Fig. 10a). Unsupervised clustering of the integrated dataset recapitulated the major immune cell populations reported in both cohorts, indicating that our experimental and analytical workflow robustly captures CSF and peripheral immune cell states in ALS4 and healthy donors (Extended Data Fig. 10a).

Single-cell analysis allowed us to distinguish both innate/innate-like (TCRγδ, MAIT, NK, myeloid) and adaptive (CD4 and CD8 T cells, and a small fraction of B cells) populations, as well as a mixed subset of *MKI67*^+^ cells (“proliferative”; Fig. 4a). Overall, we observed a trend toward an increased proportion of CD8 T cells in ALS4 patients, mirrored by a decrease in the CD4/CD8 T cell ratio compared to the healthy control (Fig. 4b).

Further sub-clustering of the myeloid compartment into classical monocytes, macrophage-like cells, cDCs, and pDCs suggested a trend toward an increased proportion of classical monocytes in patients, in parallel with our differential expression analysis in blood indicating major transcriptional changes in this innate population in ALS4 patients (Extended Data Fig. 5 and 10b,c). However, the number of CSF classical monocytes detected in our analysis was too low to support further conclusive analyses.

Focusing on the CD8 T cell compartment, we identified six subsets: Tcm CCR4⁻, Tcm CCR4^+^, Trm (*ITGAE*^+^), Tem GZMK^+^, Tem HLA-DR^+^, and Tem GZMB^+^ (Fig. 4c). Some proportional changes were observed in ALS4 patients compared to the healthy control, including an increased frequency of Tem GZMB^+^ cells -which were detected in all patients and only in two HC- and in GZMK^+^ CD8 T cells, with a decrease in Tcm CD8 T cells in ALS4 patients (Fig. 4c, Extended Data Fig. 10d).

Within the CD4 T cell compartment, we distinguished nine subsets: Tregs, terminal effector cells, IFN^+^ (*MX1*^+^), Trm CXCR6^+^ cells, as well as Tfh, Th2, Th1/Th17, and Th17 populations (Extended Data Fig. 10e,f). In contrast to the peripheral blood, the relative proportion of Th1/Th17 cells was not altered in the CSF of ALS4 patients compared to the HC, whereas we observed a decrease in Tfh cells, a trend that we did not observe in the periphery, and an increase in HLA-DR^+^ CD4 T cells. Notably, we did not observe the marked increase in cytotoxic CD4 T cells or decrease in Tregs reported in sporadic ALS^17^, further suggesting that CD4 T cell remodeling differs between sporadic ALS and ALS4 (Extended Data Fig. 10e,f).

We next performed a scTCR-seq analysis in the CSF (Fig. 4d–e, Extended Data Fig. 11). We didn’t proceed to scBCR-seq since, as expected, the number of B cells in the CSF was extremely low (Fig. 4b). Although we detected expanded TCR clonotypes in CSF from both healthy controls and patients (Extended Data Fig. 11), most control samples originated from a public dataset with substantially higher cell numbers, resulting in an imbalance in recovered cell numbers between control and ALS4 conditions. Because TCR repertoire analyses are highly sensitive to the number of recovered cells [32,33], direct comparisons between groups were challenging. Therefore, TCR repertoires were subsampled to the minimum number of recovered cells per donor. After subsampling, no differences in clonotype composition were detected between ALS4 and control samples^32,33^ (Extended Data Fig. 11).

To delineate the relationship between CNS and peripheral T cell pools, we assessed clonotype sharing between the two compartments. ALS4 patients exhibited markedly increased blood-CSF clonotype sharing within CD8 T cells relative to healthy controls, with a median of ~30% of unique CSF clonotypes detected in blood (Fig. 4d). A similar trend toward increased TCR overlap was evident for CD4 T cells, although this effect was less pronounced and did not reach statistical significance. Among all CSF CD8 T cell subsets, this difference was the most pronounced for CSF GZMK^+^ CD8 T cells (Fig. 4d). Focusing on shared clonotypes, we next investigated which CSF T cell subsets were clonally linked to which blood T cell subsets. In healthy controls, shared clonotypes were distributed across HLA-DR^+^ and Tem GZMK^+^ cells in the CSF and were linked comparably to peripheral Tem GZMK^+^ and Tem GZMB^+^ cells. In contrast, ALS4 was characterized by a predominance of CSF Tem GZMK^+^ clonotypes that mapped primarily to peripheral Tem GZMB^+^ cells (Fig. 4e). Collectively, these data indicate that ALS4 is characterized by an abnormally high frequency of CD8 T cell clonotype sharing between CSF and blood, with CSF Tem GZMK^+^ cells preferentially sharing TCRαβ sequences with peripheral Tem GZMB^+^ cells.

## Discussion

In this study, we combined single-cell transcriptomics with high-parameter spectral flow cytometry to delineate how the immune landscape evolves over the course of ALS4. Leveraging a rare cohort spanning more than six decades of disease, we profiled immune signatures in both peripheral blood and cerebrospinal fluid. Our analyses reveal that: (1) ALS4 progression is associated with a dominant CD8 T cell signature in the periphery, characterized by marked clonal expansion and GZMB expression; (2) ALS4 patients display substantial sharing of CD8 T cell clonotypes between blood and CSF, whereas such overlap is not observed in healthy controls; (3) the highest degree of clonal sharing in ALS4 patients involves CSF GZMK^+^ Tem cells, which predominantly share clonotypes with GZMB^+^ Tem cells in the blood, indicating a circulating pool of antigen-experienced CD8 T cells; and (4) peripheral CD4 T cells exhibit a Th1/Th17-skewed phenotype without comparable clonal expansion and without a prominent CSF counterpart, highlighting a discrepancy between peripheral and central CD4 T cell changes. Collectively, these findings link disease progression to coordinated peripheral and central immune remodeling and support a central role for CD8 T cells in ALS4, with implications for biomarker development and immune-based therapeutic strategies in ALS4.

Over recent decades, substantial effort has been devoted to defining whether, and by what mechanisms, the peripheral immune system -particularly T cells- contributes to neurodegenerative disorders such as ALS, which were historically considered non-immune-mediated diseases. Dissecting inflammatory pathways that underlie ALS progression is hampered by the fact that most patients receive a diagnosis only after extensive neurodegeneration has occurred, coupled with an average survival of 3-5 years that severely limits access to early immune events and reconstruction of their temporal dynamics.

ALS4 represents a rare exception to these constraints. This genetically defined, mainly familial ALS subtype, caused by highly penetrant SETX L389S variants, is characterized by juvenile onset, extremely slow progression, and minimal impact on overall life expectancy, permitting identification of affected individuals across a broad age range.

Using a *Setx* L389S knock-in mouse, we previously demonstrated that ALS4 is marked by a robust immune signature consisting of terminally differentiated, clonally expanded CD8 T cells in blood and CNS whose frequencies track disease course, and that clonally expanded CD45RA^+/dim^ CD28^−^ CD8 T cells are increased in the peripheral blood of ALS4 patients older than 59 years^12^. However, it remained unclear whether this immune signature emerges earlier in disease and whether it is mirrored within the CNS.

By extending our analyses to ALS4 patients spanning a broad range of ages and clinical stages, we now show that this CD8 T cell phenotype is not merely a late, bystander manifestation but instead arises early in disease. Elevated frequencies of CD45RA^+/dim^ CD28^−^ CD8 T cells are already detectable in young patients, and single-cell RNA/TCR sequencing reveals that clonally expanded GZMB^+^ CD8 T cells are already detectable in some individuals younger than 30 years. These cells persist as a signature that parallels clinical progression, indicating sustained, antigen-driven cytotoxic CD8 T cell responses as a defining feature of ALS4 in the periphery. Extending these analyses to CSF, we identify a concordant CD8 T cell signature in the intrathecal compartment; although limited sample size and cell numbers preclude definitive quantification of changes in specific CD8 subsets or clonal expansion relative to controls, ALS4 patients exhibit an unusually high fraction of CD8 T cells that share identical TCRαβ sequences between blood and CSF compared with controls. Both the magnitude and qualitative pattern of this sharing differ between ALS4 and healthy groups: in ALS4, GZMK^+^ CD8 T cells in CSF constitute the largest clonotypes matched to effector-memory GZMB^+^ and, to a lesser extent, GZMK^+^ cells in blood, and despite their low absolute abundance in CSF, Tem GZMB^+^ cells are disproportionately represented among clones shared with Tem GZMB^+^ and GZMK^+^ populations in the periphery. Highly expanded Tem GZMK^+^ cells in blood exhibit transcriptional and functional hallmarks of cytotoxic CD8 T cells, supporting the presence of a common pool of antigen-experienced CD8 T cells that recirculates between peripheral and intrathecal compartments in ALS4 and acquires a terminal phenotype.

In this study, we report for the first time a sporadic ALS4 case with a distinct substitution at codon 384 (E384K). Although it is difficult to draw firm conclusions from a single individual, particularly given the inherent variability among human donors (both healthy and ALS), this patient exhibits a CD8 T cell signature that parallels that observed in familial L389S ALS4 and, intriguingly, also carries the DR7.DQ2.2 haplotype. These observations highlight the need to investigate additional sporadic ALS4 cases with diverse variants.

Collectively, ALS4 emerges as a powerful model to dissect how aberrant adaptive immunity can drive motor neuron injury and suggests that the CD8 T cell component we uncover in this subtype may not be restricted to ALS4, but may instead represent a broader, under-recognized axis of T cell-mediated pathology in ALS.

Several studies have reported T cell signatures in sporadic and familial ALS^13–21^, but the findings are contrasting. Some reports describe a predominant CD4 T cell signature characterized by reduced peripheral Tregs, increased intrathecal CD4 T cell proportions and activation, or decreased peripheral Th1/Th17 subsets^14,17,21,31,34^, whereas other studies identify clonally expanded effector-memory CD8 T cells as the dominant adaptive signature in sporadic and familial ALS^19,20^. Direct comparison of these divergent results is challenging because cohorts differ in clinical and genetic composition, samples have been profiled using distinct technologies (flow cytometry, Cytometry by Time Of Flight/CyTOF, scRNA-seq), and only one study has performed paired CSF/PBMC single-cell analysis in three sporadic ALS patients, notably reporting a GZMK^+^ CD8 T cell signature with clonal expansion in CSF. Although firm conclusions remain difficult, further work delineating commonalities and differences between the CD8 T cell signature in ALS4 and in other ALS forms is clearly needed, and our data provide a framework for such investigations.

A second major question is whether ALS4 and other ALS subtypes harbor an autoimmune component. Recent reports of C9ORF72-specific CD4 T cells in the peripheral blood of ALS patients^16^, together with evidence that TDP-43 (Transactive Response DNA-binding Protein 43) mislocalization -a common pathological feature in ALS- induces splicing defects and cryptic peptides^35–38^ capable of eliciting antibody^39^ and T cell responses^40^, strongly support this possibility. In ALS4, we did not detect evidence of an antigen-specific CD4 T cell response. Our data -by arguing against a CD8 T cell clonal expansion driven by microbial infection- point toward an autoimmune-like CD8 T cell phenotype but the antigen source is yet to be determined. TDP-43 mislocalization has also been observed in ALS4 post-mortem tissues^9^, but the extent of this phenomenon and whether it is present at early disease stages when CD8 T cells begin to expand remain unknown. Furthermore, the magnitude and persistence of cytotoxic CD8 T cells and their clonal expansion contrast with the slowly progressive nature of ALS4, raising the possibility that intrinsic mechanisms (such as rapid differentiation to terminally exhausted CD8 T cells) or extrinsic factors that restrain CD8 T cell effector function are engaged.

In contrast to the pronounced CD8 T cell alterations, peripheral immune compartments were largely stable in ALS4 by spectral cytometry and scRNA-seq, despite prior reports of innate immune dysregulation-particularly in monocytes-in other ALS subtypes^6,18,41–46^. CD14^+^ monocytes in ALS4 exhibited the most prominent transcriptional changes, closely mirroring the peripheral C9orf72-associated CD14^+^ monocyte signature^19^ and suggesting convergent innate immune perturbations between these two familial forms, although our modest cohort size indicates that these findings should be viewed as supportive and warrant validation in larger studies.

This work has several limitations. First, the PBMC dataset drew on four sample sources and two sequencing batches in a nested design in which disease samples were absent from public or biobank sources, making standard batch correction approaches unsuitable for differential expression analysis. Because ALS4 progression is closely linked to aging, we prioritized inclusion of an equal proportion of age-matched controls, which prevented collection of controls from a single source; sequencing was necessarily performed in two batches dictated by patient and control availability. While this strategy strengthens the conclusion that the ALS4 CD8 T cell signature is detectable across controls of diverse origin, it increases donor-related variability and constrains the application of standard batch correction for differential gene expression. Second, the number of CSF samples from patients and controls was limited. Only five ALS4 donors consented to CSF collection; one was excluded from our analyses because of poor cell recovery, and obtaining CSF from healthy individuals is inherently challenging. As a result, we generated only one CSF control sample within our study, supplementing it with controls from public datasets. Consequently, differential expression analysis in CSF was not feasible, and differences in sequencing depth required TCR-repertoire subsampling. Third, ALS4 is a rare disease, and donor numbers were intrinsically limited, restricting statistical power; accordingly, we report nominal (unadjusted) p-values and interpret statistical associations with caution.

In conclusion, ALS4 exposes a consistent, antigen-driven CD8 T cell program that arises early, tracks clinical progression, and bridges blood and CSF while acquiring terminal cytotoxic features. To our knowledge, this is the first study to define a specific immune signature across decades of disease evolution in a form of ALS, and it opens the way to test whether a CD8 T cell-centric signature constitutes an underrecognized axis of T cell-mediated pathology in other ALS subtypes. It may also be informative to examine patients with additional *SETX* variants, including those classified as pathogenic versus of unknown significance, to better understand how alterations in the T cell program compare across mutations in *SETX*. Given that many *SETX* variants with unclear pathogenicity have been reported^11,47^, expanding analyses across a broader spectrum of mutations and their associated T cell signatures would help the field refine genotype–immune phenotype relationships. By delineating this immune trajectory, our findings provide a foundation for dissecting immune-dependent pathogenic mechanisms and for leveraging CD8 T cell signatures to stratify patients by disease course and guide personalized therapeutic interventions.

## Material and Methods

### Human participants and sample collection

Human studies were approved by the Program for the Protection of Human Subjects, Institutional Review Board of the NIH (protocol# NCT04394871), and by the Washington University in St. Louis Institutional Review Board (IRB# 201804084 and IRB# 201801081), in accordance with all relevant guidelines and regulations. All participants provided written informed consent prior to enrollment, and demographic and clinical characteristics are summarized in Supplementary Table 1. As indicated in Supplementary Table 2, PBMCs and CSF from ALS4 participants were collected at the NIH/NINDS under protocol NCT04394871. PBMCs from healthy donors were obtained at the NIH/NINDS under NCT04394871, at Washington University in St. Louis School of Medicine (IRB# 201804084), or purchased from commercial vendors (AllCells/DLS and SanguineBio).

### PBMC isolation and cryopreservation

PBMCs were isolated from peripheral blood by density-gradient centrifugation using Ficoll (Ficoll Histopaque; Sigma) or Vacutainer CPT Mononuclear Cell Preparation Tubes (BD Biosciences), according to the manufacturers’ instructions, consistent with standard human PBMC processing procedures. After isolation, PBMCs were aliquoted, frozen at −80 °C in Recovery Cell Culture Freezing Medium (Thermo Fisher Scientific), and transferred the following day to liquid nitrogen for long-term storage.

### CSF collection, cell isolation and cryopreservation

CSF was collected by lumbar puncture and processed for cell isolation as previously described^47^. Briefly, CSF was centrifuged at 300 rcf for 10 min at 4 °C to pellet immune cells; 100 μl of the cell pellet was mixed with 900 μl Recovery Cell Culture Freezing Medium (Thermo Fisher Scientific), frozen overnight at −80 °C, and transferred the following day to liquid nitrogen for long-term storage.

### Cell preparation for analysis

Frozen vials were rapidly thawed at 37 °C and cells were immediately transferred to RPMI 1640 medium (Sigma) supplemented with 2 mM L-glutamine, 10 mM HEPES, 1 mM sodium pyruvate, 1× MEM nonessential amino acids, 2.5 μM β-mercaptoethanol (all Sigma), antibiotic–antimycotic solution (Sigma), and 10% fetal bovine serum (FBS; Thermo Fisher Scientific). Cell suspensions were incubated for 2 min at 37 °C in the presence of DNase I grade II (0.2 mg/ml; Roche) prior to downstream applications to limit DNA-mediated cell aggregation.

### Flow cytometry

For surface staining, cells were resuspended at 10-16 × 10^6^ cells per ml in PBS containing 2% FBS, 2.5 mM EDTA, antibiotic–antimycotic solution, and gentamicin (Sigma), and incubated with Human TruStain FcX Fc Receptor Blocking Solution and anti-human CD16/32 blocking solution (both BioLegend) to minimize nonspecific Fc-mediated binding. Dead cells were excluded in all experiments using Live/Dead fixable viability dyes (BioLegend), and Super Bright Complete Staining Buffer (eBioscience) was used according to the manufacturer’s instructions for panels containing Super Bright fluorochromes.

#### T cell–focused staining panel.

For T cell–focused panels, we employed a sequential staining strategy for TCRγδ and chemokine receptors, adapted from previous reports^22^. Briefly, cells were first stained with anti-human TCRγδ for 10 min at 4 °C, followed by anti-human CCR5 for 5 min at 37 °C; cells were then stained with anti-human CXCR3 and anti-human CCR4 for 5 min at 37 °C, anti-human KLRG1 and anti-human CD25 for 5 min at 37 °C, and finally anti-human CCR10 for 5 min at 37 °C. All remaining surface markers (listed in Supplementary Table 2) were added as a single cocktail, incubated for 5 min at 37 °C and subsequently for 30 min at 4 °C, with Super Bright Complete Staining Buffer included per the manufacturer’s recommendations.

#### General immunophenotyping panel.

For general immunophenotyping, cells were stained with anti-human TCRγδ for 10 min at 4 °C, followed by anti-human CD25 for 10 min at 4 °C. The remaining markers (see Supplementary Table 2) were added as a cocktail and incubated for 30 min at 4 °C, with Super Bright Complete Staining Buffer included according to the manufacturer’s protocol. For biotinylated primary antibodies, Qdot 605 Streptavidin Conjugate (BioLegend; 1:500) was used.

#### Intracellular staining and data acquisition.

For intracellular staining, cells were fixed and permeabilized using the eBioscience Intracellular Fixation & Permeabilization Buffer Set (eBioscience) according to the manufacturer’s instructions. Flow cytometry data were acquired on a 5-laser Cytek Aurora spectral cytometer (Cytek Biosciences) with a SpectroFlo software version 3.3, and data were analyzed using Cytobank software version 10.7 (Cytobank) and FCS Express 7 (De Novo Software) in accordance with current recommendations for high-dimensional cytometry.

### Fluorescence-Activated Cell Sorting

For PBMC cell sorting followed by single cell-RNA/TCR/BCRseq, cell suspensions were incubated with Human TruStain FcX Fc Receptor Blocking Solution and anti-human CD16/32 blocking solution (BioLegend), followed by staining with TotalSeq-C hashtag antibodies (BioLegend, 1:500) followed by three washes. For total PBMC sorts, cells were stained with Zombie Fixable Viability Kit (BioLegend), and live (Zombie-negative) cells were collected. For sequencing of sorted CD8 T cells, cells were stained with biotinylated anti-TCRγδ (Clone: B1, 331206, Biolegend. 1:200), biotinylated anti-CD19 (Clone: H1B19, 302204, Biolegend, 1:200) and biotinylated anti-Vα7.2 (Clone: 3C10, 351724, Biolegend, 1:200), followed by streptavidin-FITC (405201, Biolegend, 1:500), together with anti-CD4 PECy7 (SK3, 344611, Biolegend, 1:400) and anti-CD8 PE (Clone: SK1, 344705, Biolegend, 1:400); stained cells were sorted on a FACSAria III or Symphony S6 (BD Biosciences).

### ELISA for CMV-specific IgG and avidity

Serum samples from 17 participants (Supplementary Table 1) were analyzed for CMV-specific IgG and IgG avidity using the SERION ELISA IgG Classic and the SERION ELISA Avidity Reagent Kit (Virion, Germany), according to the manufacturer’s instructions. Optical density (OD) values were recorded, converted to antibody units (U/ml) using the manufacturer’s calibration curve, and interpreted within the validated OD range of 0.42–1.43; values <0.42 were considered negative. The avidity index (%) was calculated as specified by the manufacturer, with an avidity index >55% indicating past CMV infection (>3 months), 45–55% consistent with primary infection approximately 1–3 months prior, and <45% indicative of acute primary CMV infection.

### Single cell-RNA/TCR/BCR sequencing

For PBMCs, pools of cells labeled with TotalSeq-C hashtag antibodies (Biolegend) were resuspended in PBS containing 0.04% BSA at a final concentration of 1,600 cells/μl. For CSF samples, cells were resuspended directly in 20 μl. Cell suspensions were then subjected to droplet-based, massively parallel single-cell RNA sequencing using the Chromium X system (10x Genomics), following the manufacturer’s instructions, at the Washington University Genome Technology Access Center (GTAC). For PBMCs, cells were loaded to achieve a targeted recovery of 20,000 cells per run. 5′ gene expression (5′-GEX) libraries were prepared by GTAC and sequenced on NovaSeq X Plus flow cells (Illumina), targeting 1 billion read pairs per library for PBMC samples and 500 million read pairs per library for CSF samples.

### Single-cell data processing

Single-cell libraries were processed in two sequencing batches. The first batch comprised PBMC, sorted CD8 T-cell, and CSF samples corresponding to three age groups and was processed using the *Cell Ranger multi* pipeline v8.0.1 (available at the 10x website). The second batch included two additional PBMC cohorts spanning multiple age groups and one additional CSF donor and was processed using *Cell Ranger count* and *vdj* v8.0.1. Gene expression libraries were aligned to the GRCh38 reference (refdata-gex-GRCh38-2024-A), and V(D)J libraries were processed using refdata-cellranger-vdj-GRCh38-alts-ensembl-7.1.0 reference.

Raw sequencing data from the public dataset Wang et al.^23^ was downloaded from GSE243905 and PRJNA717310 NCBI repository. For PBMC samples, only the first replicate was used. Fastq files were processed using *Cell Ranger count* and *vdj* v9.0.1 with the same reference genomes as stated above.

To resolve pooled donors within each sorted sample, genotype-based demultiplexing was performed using *Souporcell*^48^ v2022.12. For each Cell Ranger output, the aligned BAM file and the corresponding cell barcode file were used as inputs. The number of expected genotype clusters was set according to the number of donors pooled in the corresponding sample. Only cells classified as genotype singlets were retained for downstream analysis. Final donor identities were assigned by combining genotype-based demultiplexing with HTO signal. Souporcell genotype clusters were first inspected to identify groups of cells corresponding to individual donors. These genotype clusters were then matched to the expected experimental donor identities using the dominant normalized HTO signal detected in each cluster.

Filtered Cell Ranger matrices were imported into the *R*^49^ environment v4.3.0. Gene expression counts were used to initialize Seurat objects using *Seurat*^50^ package v4.3.0. PBMC, sorted CD8 T cell, and CSF samples were processed for downstream analysis as three separate Seurat objects. Gene expression counts were normalized using log-normalization with a scale factor of 10,000. Each dataset was analyzed using a hierarchical, stepwise clustering strategy. After initial clustering, cells were grouped into major immune populations (T cells, NK cells, myeloid cells, and B cells). Each major population was then subsetted and reanalyzed independently by repeating the standard clustering workflow to resolve progressively finer cell subsets. For each clustering step, 1500 highly variable genes were selected. To prevent T-cell or B-cell receptor genes from driving dimensionality reduction and clustering, genes beginning with TRA, TRB, IGH, IGK, IGL were removed from the variable feature set before scaling and PCA from the objects where T or B cells were present. The data were scaled using the selected variable features. During scaling, total UMI count (nCount_RNA) and mitochondrial percentage (percent.mt) were regressed out. Principal component analysis was performed on the scaled expression matrix using the selected variable genes. Harmony integration (*harmony*^51^ package v0.1.1) was then applied to the PCA embeddings to correct for donor and sequencing batch-associated effects.

UMAP dimensionality reduction was performed using the first 15 Harmony dimensions. The same Harmony dimensions were used to construct the nearest-neighbor graph. Graph-based clustering was performed in Seurat using the shared nearest-neighbor graph. For the CSF CD4 T cell, CD8 T cell, and myeloid objects, donor integration was performed using Seura’s reciprocal PCA (rpca) integration workflow due to the substantial imbalance in cell numbers across donors. In comparison with harmony, rpca yielded improved integration for these objects while maintaining biologically meaningful population structure. For rpca integration, each dataset was split by donor, and highly variable genes were identified independently for each donor using the variance-stabilizing transformation method with 3,000 features. T-cell receptor and B-cell receptor genes were removed from the variable feature set. Integration features were then selected across donors using *SelectIntegrationFeatures* with 2,000 features. Each donor-specific object was scaled and subjected to PCA using the selected integration features. Integration anchors were identified across donors using rpca method with the first 30 principal components, followed by data integration. The integrated assay was then scaled while regressing out total RNA counts and mitochondrial gene percentage. PCA was performed on the integrated data, and the first 15 principal components were used for UMAP visualization, nearest-neighbor graph construction, and clustering.

Quality control was performed at the clustering level using multiple metrics, including the total number of detected UMIs, the number of detected genes, and the percentage of mitochondrial reads. Cells with low UMI counts or low numbers of detected genes were removed as low-quality cells. Cells with high mitochondrial content were removed to exclude damaged or dying cells. Cells with excessively high numbers of detected genes or UMIs were removed to reduce the contribution of multiplets or other technical artifacts. Cells with a high level of expression of more than one cell population-specific marker were considered as doublets and removed. Blood immune cells were annotated to match populations from Terekhova et al.^22^ immune cell atlas reference. For the CSF dataset, clustering resolution was chosen based on the identification of biologically meaningful populations supported by canonical marker gene expression.

One ALS4 donor was excluded from the CSF dataset because only 101 cells passed quality control, which was considered insufficient for reliable downstream analyses. One public healthy control sample was excluded from the CSF dataset because of an abnormally high proportion of naive T cells, consistent with suspected peripheral blood contamination.

### Single-cell TCR repertoire analysis

T cell receptor repertoire analysis was performed using Cell Ranger V(D)J filtered contig annotations. Contigs were retained if they were assigned to cells from GEX T cells and annotated as high-confidence, productive, and full-length. Only cells with exactly one productive TRA chain and one productive TRB chain were retained for downstream analyses. Clonotypes were defined by concatenating CDR3 nucleotide sequence, V gene, J gene from both alpha and beta chains. Clonal expansion was quantified by calculating the number of cells assigned to each clonotype within each donor and cell population. For each donor, clonal inequality was quantified using the Gini coefficient with the *DescTools*^52^ package v0.99.54 within each annotated T cell subset. In CSF, to account for differences in CD8 T cell numbers across donors, clonal inequality was assessed using a subsampling strategy. For each permutation, the same number of CD8 T cells was randomly sampled from each donor, using the minimum donor cell count as the subsampling depth. This procedure was repeated 100 times.

For each subsampling iteration, clonotype sizes were calculated within each donor and CD8 T cell population, and clonal inequality was quantified using the Gini coefficient. Final donor-level values were summarized as the mean Gini coefficient across 100 permutations.

To characterize repertoire occupancy, clonotypes were grouped by clone size into small (≤5 cells), medium (>5 and ≤20 cells), large (>20 and ≤100 cells), and hyperexpanded (>100 cells) categories. For each donor, disease group, age group, and major immune population, the percentage of cells occupied by each clone-size category was calculated. Mean repertoire occupancy was then summarized across donors and visualized as stacked bar plots stratified by disease group and age group.

To assess clonal relationships between CD4 Terminal effector cells and other CD4 T cell subsets, the number of unique paired αβ clonotypes from each Terminal effector subset that were also detected in other CD4 T cell subsets was determined. Clonal overlap was expressed as the percentage of unique clonotypes in the reference Terminal effector subset that were shared with each comparison subset. Clonotypes detected exclusively within a Terminal effector subset and absent from all other CD4 T cell subsets were classified as ‘Terminal effector-specific’.

To quantify the overlap between blood and CSF T cell repertoires, unique clonotypes were identified separately in PBMC and CSF samples for each donor and major T cell population (CD4 T cells and CD8 T cells). For each donor, the proportion of unique CSF clonotypes that were also detected in the matched PBMC sample was calculated by dividing the number of shared clonotypes by the total number of unique CSF clonotypes. Repertoire overlap was compared between disease groups separately for CD4 T cells and CD8 T cells. The analysis was performed both at the level of total CSF CD4 and CD8 T cells and separately for individual CD8 T cell subpopulations.

To visualize clonal overlap between CSF and PBMC CD8 T cell subsets, PBMC CD8 T cell annotations were grouped into Temra, HLA-DR^+^, Tem GZMB^+^, Tem GZMK^+^, and Other subsets. For each donor, clonotypes detected in both CSF and PBMC were identified by matching paired αβ clonotypes across tissues. Shared clonotypes were summarized according to their CSF and PBMC annotations. Within each disease group, percentages were calculated relative to the total number of shared CSF-PBMC clonotypes, such that the proportions of CD8 T-cell subsets summed to 100% independently for the CSF and PBMC compartments.

Public TCR clonotypes (ones that are shared between multiple donors in current study) were identified by comparing either paired αβ TCR clonotypes or β TCR clonotypes across donors within unsorted PBMC, sorted CD8 T cells or CSF. For clonotype, the number of donors sharing the same clonotype was calculated. Clonotypes detected in more than one donor were classified as public TCR clonotypes. For each public clonotype, the contributing donors and corresponding disease groups were recorded.

To annotate antigen-specific T-cell receptors, the curated VDJdb database was downloaded from the VDJdb database^53^. VDJdb entries were filtered to retain records with available CDR3 sequences and epitope annotations. Blood-related CDR3β amino acid sequences from current study (CD4 T, CD8 T, both sorted and unsorted) were then matched to VDJdb CDR3β sequences. For each donor, the number and percentage of unique TCRβ clonotypes matching VDJdb entries were calculated relative to the total number of unique TCRβ clonotypes. Matching clonotypes were annotated using the reported antigen specificity and associated metadata available in VDJdb.

### Single-cell BCR repertoire analysis

B cell receptor repertoire analysis was performed using filtered contig annotations generated by the Cell Ranger V(D)J pipeline. Cells were retained for downstream analysis only if they contained exactly one productive heavy chain and one productive light chain and had GEX annotation from B cells. Filtered donor-specific contig annotation files were reformatted into the standard 10x Genomics V(D)J output structure and loaded into R using the *immunarch*^54^ package v0.9.1. BCR sequence similarity was calculated using Hamming distance based on CDR3 nucleotide sequences. Clonal clusters were then defined using seqCluster with a 90% sequence similarity threshold and the same V and J genes. For each donor, clonal inequality was quantified using the Gini coefficient with the *DescTools*^7^ package v0.99.54 within each annotated B cell subset.

### HLA typing

For HLA typing, we used data generated in the current study and public controls from Wang et al.^23^, as well as sporadic ALS samples from Itou et al.^30^. Raw data from Itou et al.^30^ was downloaded from GSE244263, from Campisi et al.^12^ was downloaded from GSE180410 and processed using *Cell Ranger count* v9.0.1 with the GRCh38 reference genome. HLA typing was performed from donor-specific bam files generated from the single-cell RNA-seq alignment output. For each donor, cell barcodes were used to subset reads from the Cell Ranger bam file. Reads were retained if they contained a matching CB:Z cell barcode tag. Donor-specific BAM files were then converted to BAM format, sorted, and indexed using *samtools*^55^ v1.23.1. HLA genotypes were inferred from the donor-specific BAM files using *arcasHLA*^56^ v0.5. First, HLA-derived reads were extracted in single-end mode using the default fragment length. Second, genotyping was performed across all supported HLA loci. Genotype calls were exported as JSON files and parsed in R using the *jsonlite*^57^ package v1.8.4. For downstream analysis, inferred alleles were converted into a donor-level table. Donors were then assigned to predefined HLA haplotype groups based on the presence of specific allele combinations, including DQ2.5, DQ2.2, DQ8, DQ7, DQ9, DR3-DQ2.5, DR4-DQ8, DR7-DQ2.2, DP2.1, DP4, and DP5. Donors without any matching predefined combination were labeled as ‘No matching group’.

### Differential expression analysis

#### High vs low-expanded clones

For differential expression high vs low-expanded clones for CD8 T cells, clonotypes with ≤5 cells were classified as low-expanded, whereas clonotypes with >20 cells were classified as high-expanded; clonotypes with intermediate expansion were excluded from this comparison. Differential expression between high- and low-expanded CD8 T cells was then performed within each donor for each CD8 T cell subpopulation using the Wilcoxon rank-sum test from *presto*^58^ package v1.0.0. TCR, BCR, mitochondrial, and ribosomal genes were excluded from testing. To assess the reproducibility of donor-level differential expression results, genes were considered consistent if they were significantly differentially expressed in the same direction in at least two donors. Only the Temra, Tem GZMK^+^, and Tem GZMB^+^ subsets met these criteria. For these populations, donor-level differential expression results were combined by random-effects meta-analysis using the *metafor*^59^ package v5.0-1. Standard errors were estimated from the nominal p-value and log-fold change, and effect sizes were combined using restricted maximum-likelihood estimation. Multiple testing correction was performed using the Benjamini-Hochberg method.

#### ALS4 vs healthy controls

Differential gene expression analysis between ALS4 and healthy controls for immune subpopulations was performed using a pseudobulk approach. Analysis was restricted to NIH PBMC samples from the first sequencing batch. For each annotated cell population, raw gene expression counts were aggregated by donor using Seura’s *AggregateExpression* function. Donor-level pseudobulk count matrices were then analyzed using *edgeR*^60^ v3.42.4 and *limmavoom*^61^ workflow v3.56.1. Genes with low expression were removed using *filterByExpr* function, and library normalization factors were estimated using the trimmed mean of M-values method. These normalization factors were incorporated during mean-variance modeling with voom, followed by linear modeling with disease status as the predictor variable. Differential expression between ALS4 and control donors was assessed using empirical Bayes moderation. Differentially expressed genes were defined using an adjusted p value < 0.05 threshold.

Gene set enrichment analysis for classical monocytes was performed using the *fgsea*^62^ package v1.26.0 with the Hallmark gene sets from the *msigdbr*^63^ package v7.5.1. Genes were ranked by the t-statistic from the differential expression analysis. For comparison with the published C9 ALS monocyte signature from Zhang et al.^19^, the gene signature was obtained from Source Data corresponding to Figure 1 (1i_CD14_C9_vs._fHC). The top 100 genes ranked by average log2fold change were used as the query gene set and tested for enrichment against the ranked gene list from the present study and vice versa.

For overall pseudobulk gene expression PCA visualization, all donors were included. Gene expression counts from the PBMC object were aggregated at the donor level. Library normalization factors were estimated using the trimmed mean of M-values method, and log-transformed counts per million (logCPM) were calculated with a prior count of 1. Principal component analysis was then performed on the transposed donor-level logCPM matrix using *prcomp* function from *stats*^49^ package v4.3.0 with centering and scaling enabled.

### Visualization

Bar plots, pie charts, scatter plots, density plots, and volcano plots were generated using *ggplot2*^64^ v3.4.2. UMAP projections and dot plots were generated using *Seurat*^50^ v4.3.0. Rasterization of high-density single-cell plots was performed using *ggrastr*^65^ v1.0.1. UMAP density plots were visualized by performing two-dimensional kernel density estimation separately for each disease and age group using the *kde2d* function from *MASS*^66^ package v7.3-58.4 with a 200 × 200 evaluation grid. Each cell was assigned the density value of the nearest position on the corresponding kernel density grid for downstream visualization. Gene labels for volcano plots were generated using *ggrepel*^67^ v0.9.3. Alluvial bar plots were generated using *ggalluvial*^68^ v0.12.5. PCA of immune cell composition was performed using a donor-by-subpopulation matrix generated by concatenating the proportions of all annotated immune cell subsets (B cells, CD4 T cells, CD8 T cells, γδ T cells, NK cells, and myeloid cells) for each donor. PCA was performed using the *prcomp* function from *stats*^3^ package v4.3.0 with centering and scaling enabled.

## Extended Data

**Extended Data Figure 1.**
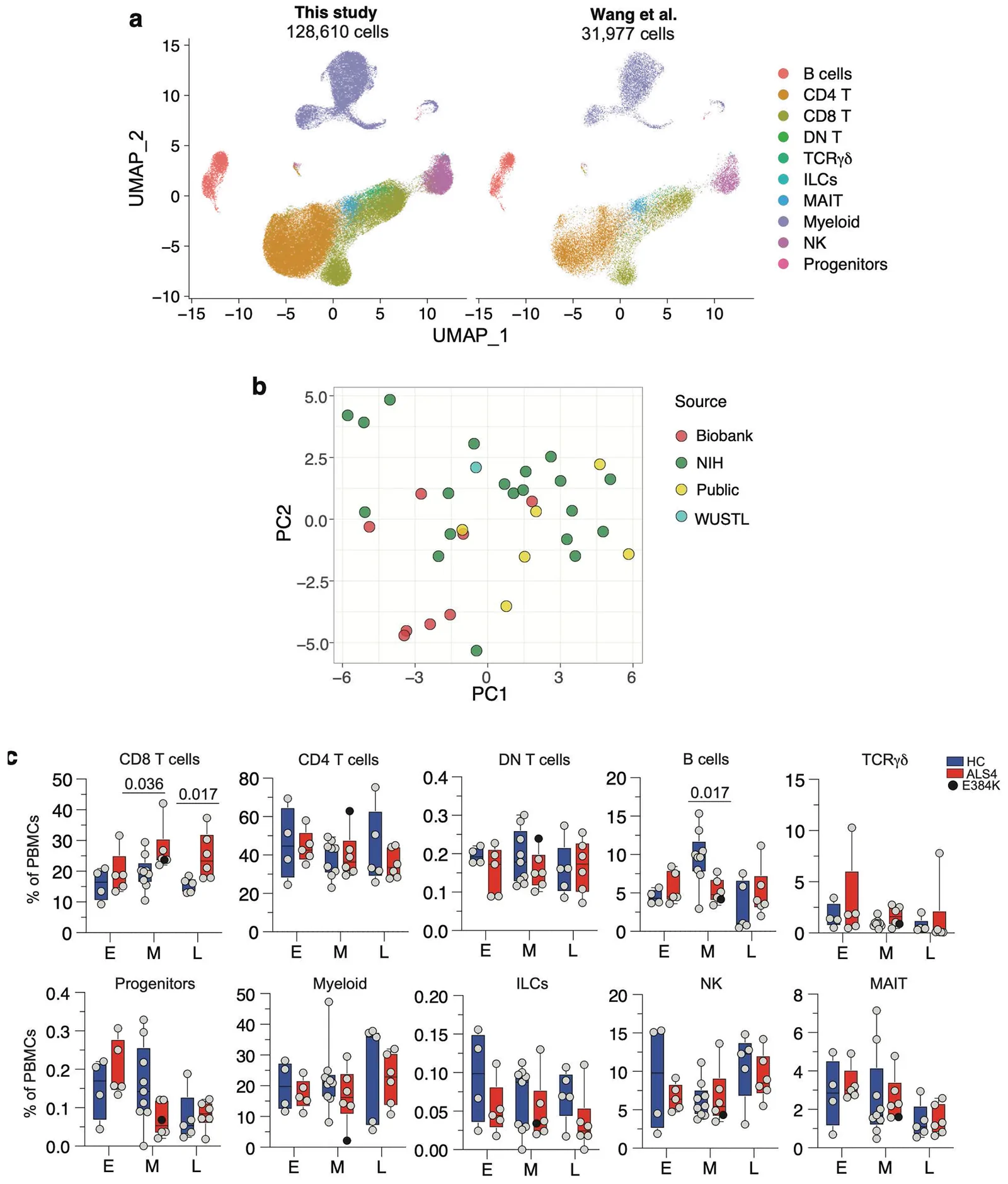
Related to Figure 1. Single-cell RNA sequencing of PBMCs from healthy controls (HC) and ALS4 patients. **a.** Uniform manifold approximation and projection (UMAP) of PBMCs from ALS4 patients and HC profiled in this study (**left**), together with PBMCs from six additional healthy controls reanalyzed from Wang et al., JCI Insight 2024 (**right**), with all cells colored by cluster identity. **b**. Principal component analysis based on proportional distribution of all PBMC subpopulations (Methods), colored by the source of the sample. Each dot represents a sample. **c.** Boxplots of the relative distribution of the indicated subsets within the PBMCs. Black circle: ALS4 patient carrying the E384K variant. ALS4, n=17: E, n=5; M, n=6; L, n=6; HC, n=18: E, n=4; M, n=9; L, n=5. Boxplot hinges indicate the 25th and 75th percentiles, whiskers extend from minimum to maximum values, all data points are shown, and horizontal bars denote the median. p-value calculated using a Mann-Whitney test.

**Extended Data Figure 2.**
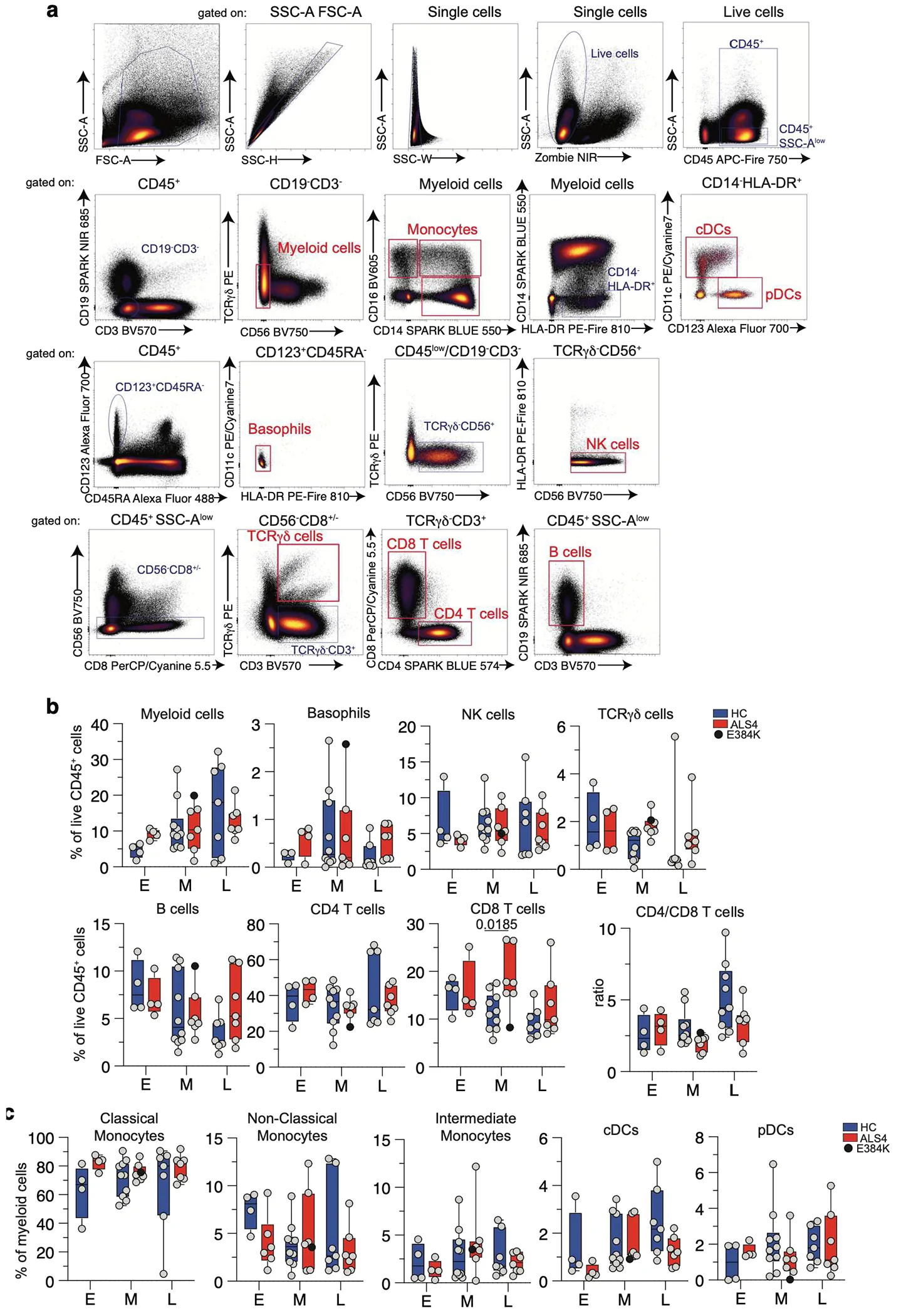
Peripheral immune landscape of ALS4 patients assessed by spectral flow cytometry. PBMCs from HC and ALS4 patients were profiled by spectral flow cytometry. **a.** Gating strategy for major immune lineages and subsets; the parent population for each flow cytometry plot is indicated above the corresponding panel. **b**,**c**. Boxplots of the relative distribution of the indicated subsets in total PBMCs (**b**) and within the myeloid compartment (**c**) across HC and ALS4 groups. Black circle: ALS4 patient carrying the E384K variant. ALS4, n=18: E, n=4; M, n=7; L, n=7; HC, n=21: E, n=4; M, n=10; L, n=7. Boxplot hinges indicate the 25th and 75th percentiles, whiskers extend from minimum to maximum values, all data points are shown, and horizontal bars denote the median. p-value calculated using a Mann-Whitney test comparing ALS4 and control donors within each age group.

**Extended Data Figure 3.**
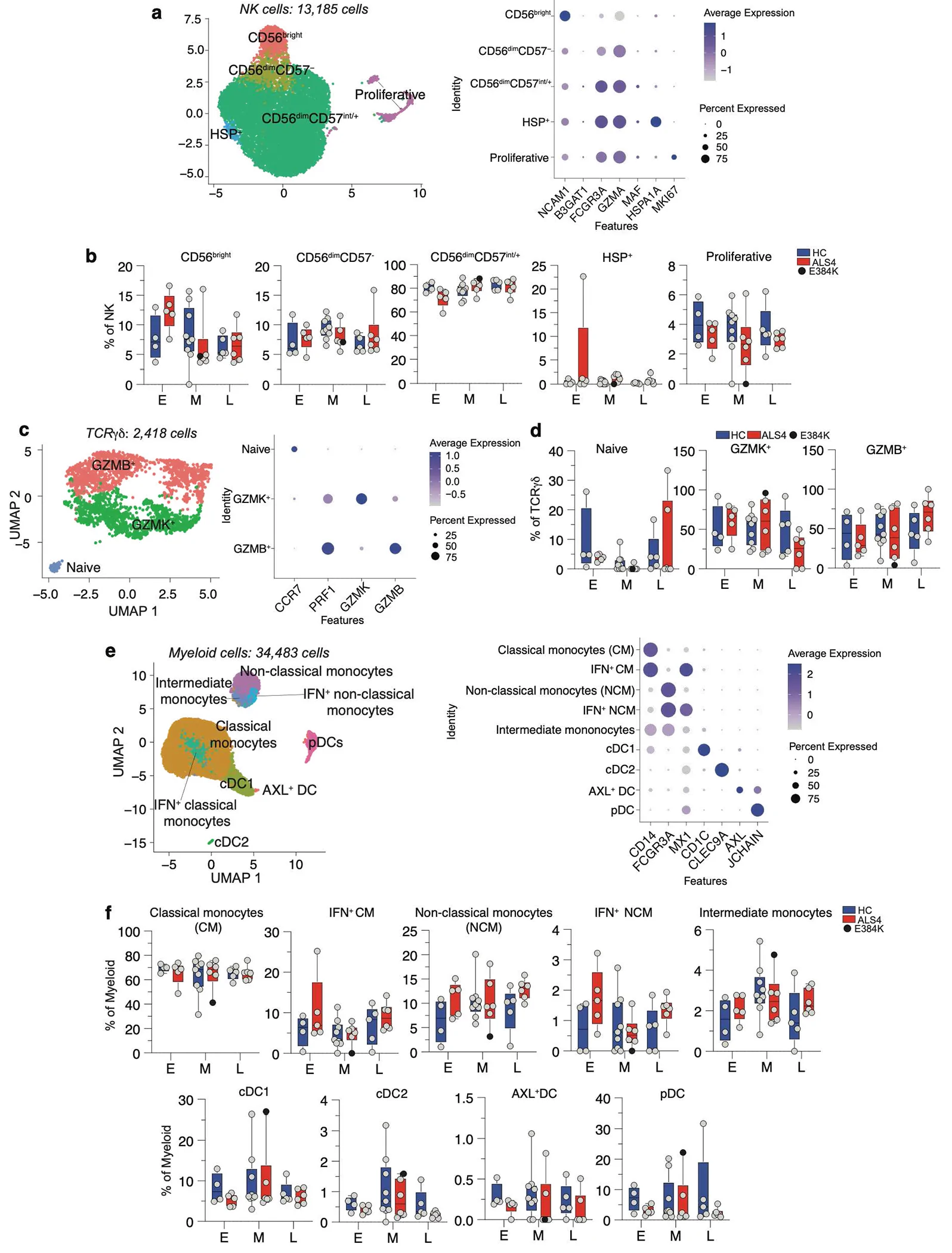
Peripheral distribution of innate immune cells is preserved during ALS4 progression. NK (**a**-**b**), TCRγγT cells (**c**-**d**) and myeloid cells (**e**-**f**) in PBMCs from HC and ALS4 patients were profiled by scRNA-seq. **a**, **c**, **e. Left**, UMAP projection of the indicate subsets colored by cluster identity. **Right**, dot plots showing expression of canonical marker genes used to delineate NK (**a**), TCRγδ (**c**) and myeloid (**e**) cell subsets. **b**, **d**, **f**. Boxplots of the relative distribution of the indicated subsets within NK (**b**), TCRγδ (**d**) and myeloid (**f**) compartments. Black circle: ALS4 patient carrying the E384K variant ALS4, n=17: E, n=5; M, n=6; L, n=6; HC, n=18: E, n=4; M, n=9; L, n=5. Boxplot hinges indicate the 25th and 75th percentiles, whiskers extend from minimum to maximum values, all data points are shown, and horizontal bars denote the median.

**Extended Data Figure 4.**
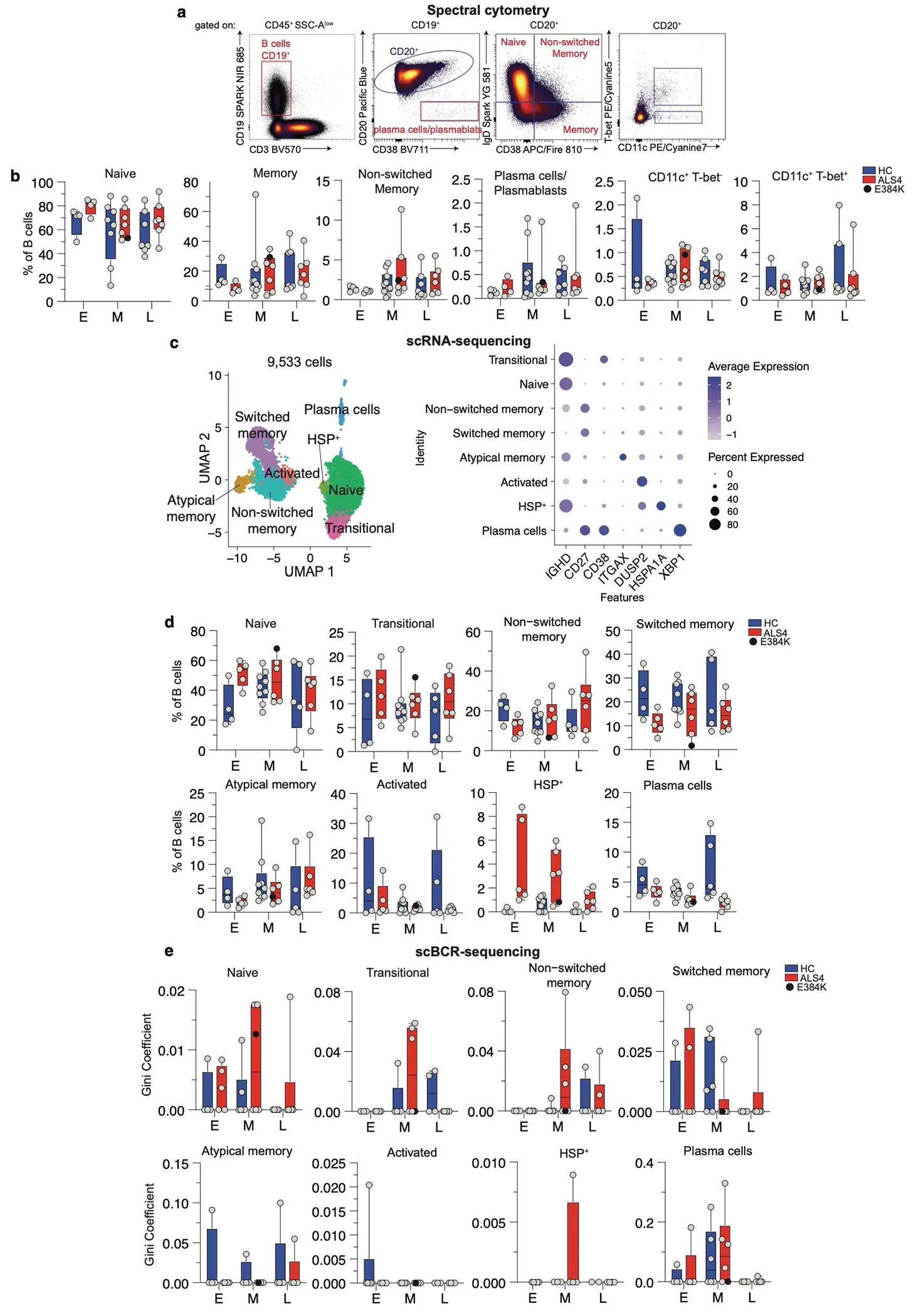
The distribution and clonal expansion of peripheral B cells is not altered during ALS4 progression. Peripheral B cells from HC and ALS4 patients were profiled by spectral flow cytometry (**a-b**) and scRNA/BCR sequencing (**c-e**). **a.** Gating strategy for B cell subsets; the parent population for each flow cytometry plot is indicated above the corresponding panel. **b**. Boxplots of the relative distribution of the indicated subsets within the B cell compartment across HC and ALS4 groups. Black circle: ALS4 patient carrying the E384K variant. ALS4, n=18: E, n=4; M, n=7; L, n=7; HC, n=21: E, n=4; M, n=10; L, n=7. **c. Left**, UMAP projection of the indicate B cell subsets colored by cluster identity. **Right**, dot plots showing expression of canonical marker genes used to delineate B cell subsets. **d.** Boxplots of the relative distribution of the indicated subsets within the B cell compartment. **e.** Gini coefficients quantifying BCR repertoire inequality for the indicated B cell subsets. Black circle: ALS4 patient carrying the E384K variant. ALS4, n=17: E, n=5; M, n=6; L, n=6; HC, n=18: E, n=4; M, n=9; L, n=5. Boxplot hinges indicate the 25th and 75th percentiles, whiskers extend from minimum to maximum values, all data points are shown, and horizontal bars denote the median.

**Extended Data Figure 5.**
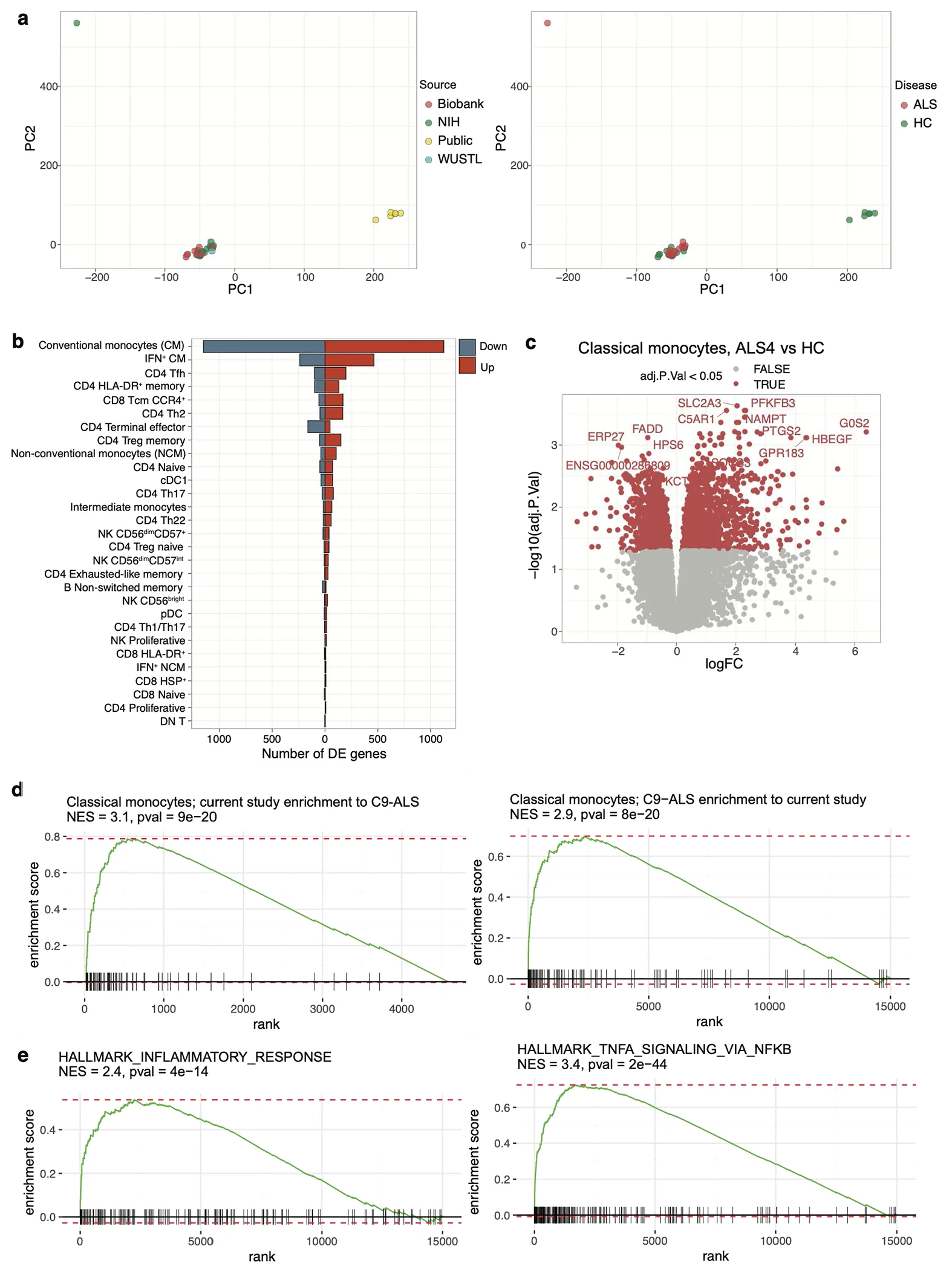
Differential gene expression in classical monocytes from ALS4 patients and a subset of HC suggests a signature shared with C9-ALS. **a.** Principal component analysis (PCA) of pseudobulk gene expression in the total PBMCs across all samples, colored by sample origin (**left**) or by disease condition (**right**). **b-d**. Differential gene expression (DE) analysis comparing PBMCs pseudobulk gene expression from ALS4 samples (n= 9) with HC (n=3). Only NIH samples from the same sequencing batch are used. **b**. Number of DE by cell subsets. **c**. Volcano plots displaying genes significantly up- or downregulated in classical monocytes from ALS4 versus healthy donors. **d**. Gene enrichment between the ALS4 classical monocyte DE signature and the classical monocyte transcriptional program reported in C9-ALS by Zhang et al., Nature Neuroscience 2026. **e**. Hallmark collection selected pathway enrichment to ALS4 classical monocyte DE signature.

**Extended Data Figure 6.**
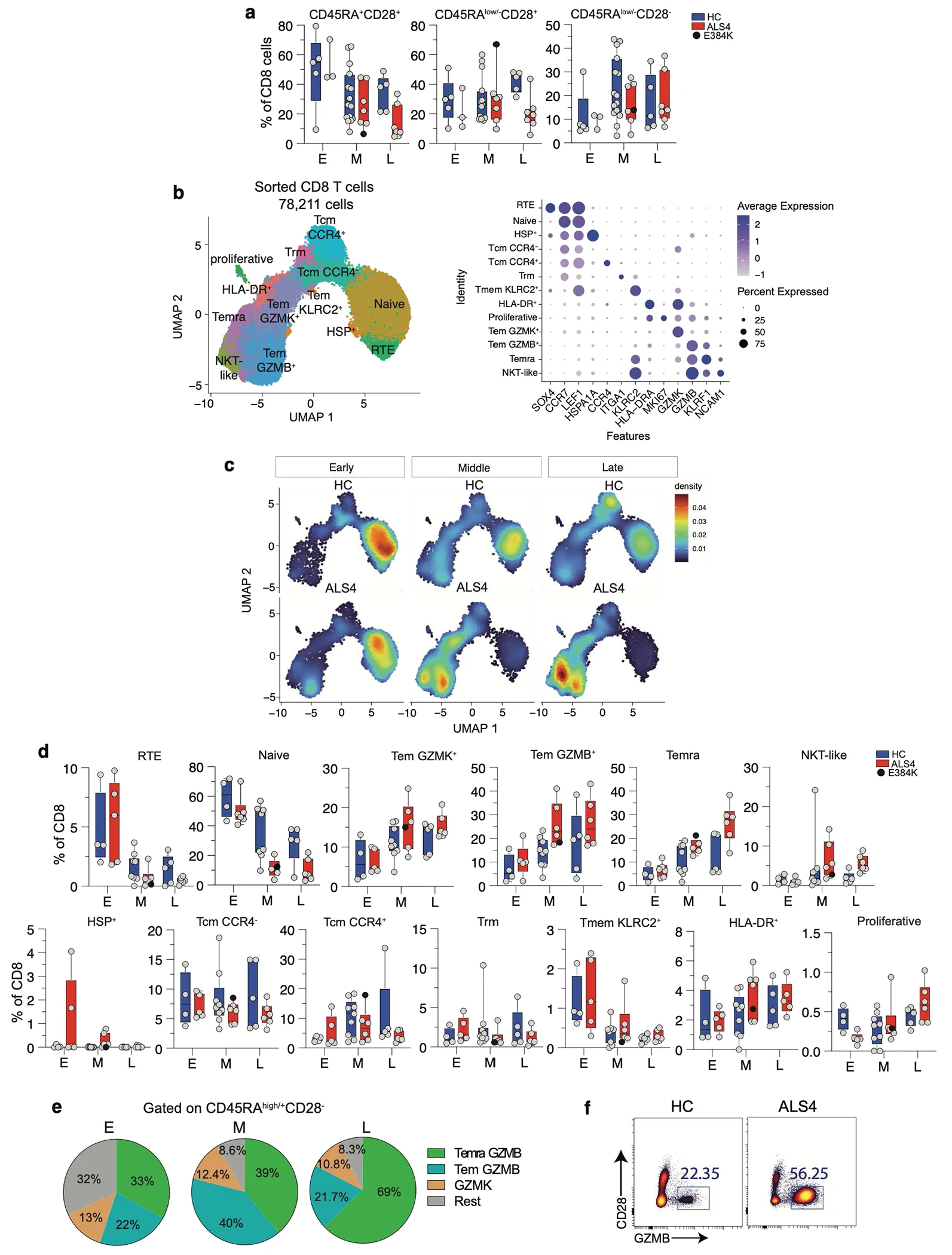
Related to Figures 1d and 2a–c. Integrated spectral flow cytometry and scRNA-seq analysis of peripheral CD8 T cells in ALS4. **a.** CD8 T cells in PBMCs were profiled by spectral flow cytometry after gating on CD14^−^ CD19^−^ TCRγδ^−^ TCR Vα7.2^−^ CD56^−^ CD3^+^ CD8^+^ cells. Boxplots show relative frequencies of the indicated subsets defined by CD45RA and CD28 expression in ALS4 patients (n=17: E, n=3; M, n=7; L, n=7) and HC (n=25: E, n=5; M, n=15; L, n=5). Black circle: ALS4 patient carrying the E384K variant. **b**-**c**. CD8 T cells were sorted from total PBMCs and analyzed by scRNA-seq. **b**. **Left**, UMAP projection of the indicate CD8 T cell subsets colored by cluster identity. **Right**, dot plots showing expression of canonical marker genes used to delineate CD8 T cell subsets. **c**. UMAP density plots of the relative distribution of CD8 T cell subsets across HC and ALS4 groups and age strata. n= 9 ALS4 and 9 HC: E, n=3; M, n=3; L, n=3 per group. **d.** Boxplots of the relative frequency of CD8 T cell subsets within PBMCs. For donors profiled by both total PBMC and sorted CD8 T cell sequencing, the corresponding cell proportions were averaged. Black circle: ALS4 patient carrying the E384K variant. ALS4, n=17: E, n=5; M, n=6; L, n=6; HC, n=18: E, n=4; M, n=9; L, n=5. Boxplot hinges indicate the 25th and 75th percentiles, whiskers extend from minimum to maximum values, all data points are shown, and horizontal bars denote the median. **e**. Pie chart of the relative proportion of GZMB^+^ and GZMK^+^ CD8 T subsets within CD45^high/+^CD28^−^ CD8 T cells in ALS4 patients as determined by spectral flow cytometry. E, n=5; M, n=6; L, n=6. **f.** Dot plots showing the frequency of GZMB^+^ CD28^−^ CD8 T cells in two siblings, one carrying the ALS4 mutation (ALS4, 8 years old) and one non-carrier (HC, 10 years old).

**Extended Data Figure 7.**
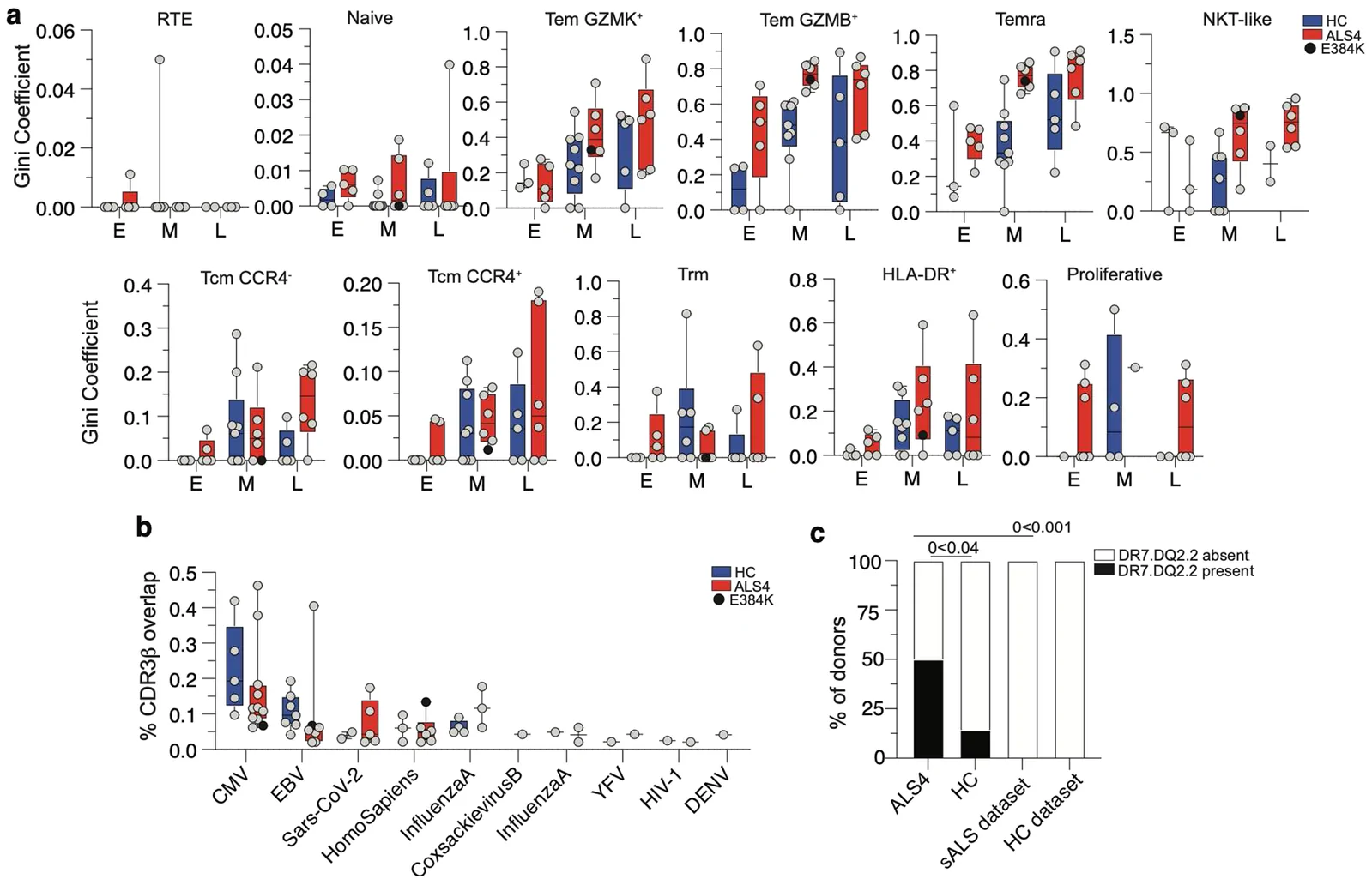
Analysis of peripheral CD8 T cell clonality and HLA haplotype. **a.** Gini coefficients quantifying TCR repertoire inequality for the indicated peripheral CD8 T cell subsets. Black circle: ALS4 patient carrying the E384K variant. ALS4, n=17: E, n=5; M, n=6; L, n=6; HC, n=18: E, n=4; M, n=9; L, n=5. **b**. Frequency of CDR3β clonotypes with matches in the public VDJdb database. Black circle: ALS4 patient carrying the E384K variant. Boxplot hinges indicate the 25th and 75th percentiles, whiskers extend from minimum to maximum values, all data points are shown, and horizontal bars denote the median. **c.** Presence of the DR7-DQ2.2 haplotype in ALS4 patients and controls, summarizing data from this study (ALS4, n=17; HC, n=12), Campisi et al., Nature 2022 (ALS4, n=3; HC, n=3), Wang et al., JCI Insight 2024 (ALS4, n=5) and Itou T et al., Journal of Neuroinflammation 2024 (HC, n=10; sporadic ALS, n=30). p-values are calculated using Fisher’s exact test.

**Extended Data Figure 8.**
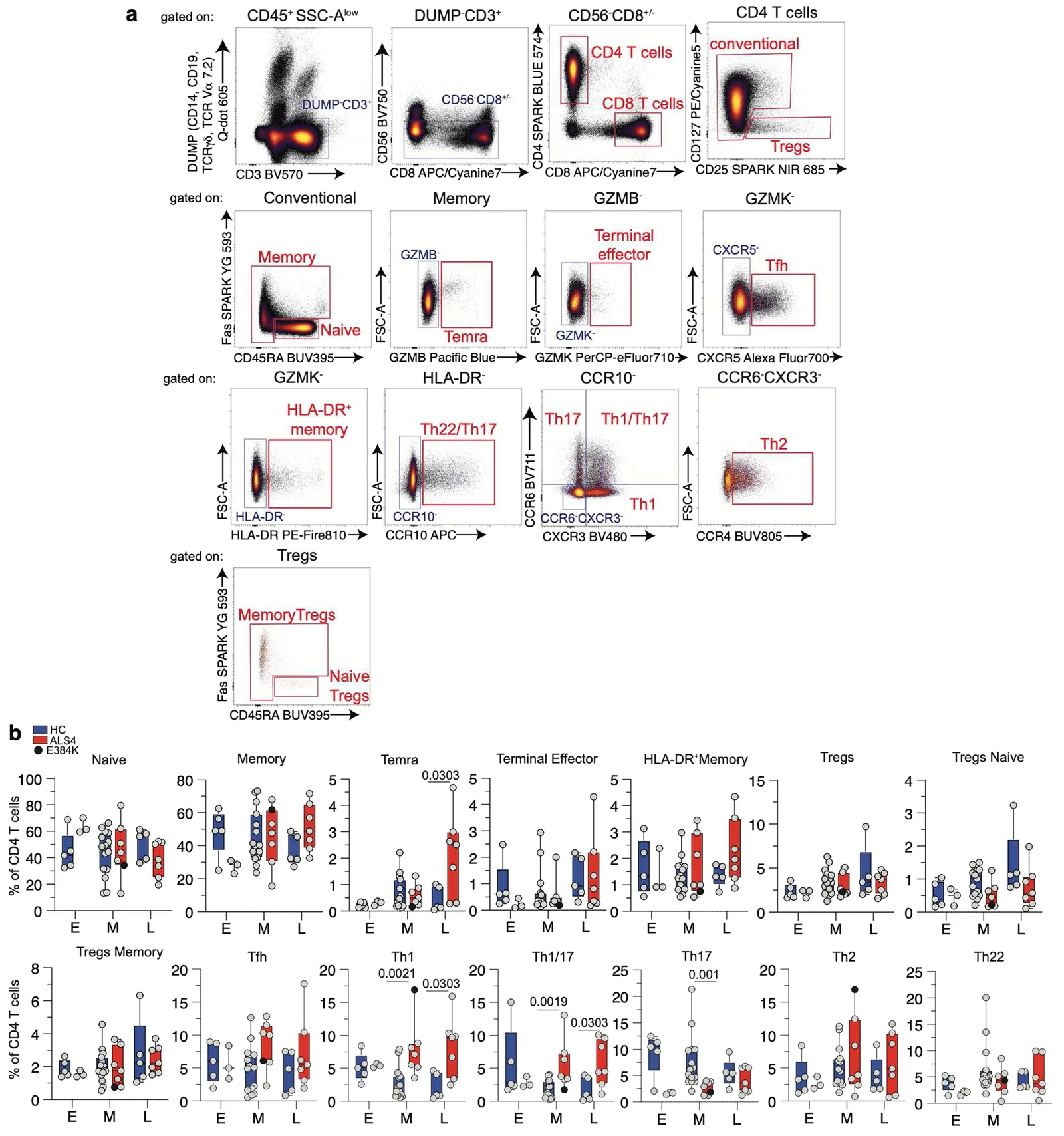
Related to Fig. 3a. Analysis of peripheral CD4 T cells by spectral flow cytometry. Peripheral CD4 T cells from HC and ALS4 patients were profiled by spectral flow cytometry. **a.** Gating strategy for CD4 T cell subsets; the parent population for each flow cytometry plot is indicated above the corresponding panel. **b**. Boxplots of the relative distribution of the indicated subsets within the CD4 T cell compartment across HC and ALS4 groups. Black circle: ALS4 patient carrying the E384K variant. ALS4, n=17: E, n=5; M, n=6; L, n=6; HC, n=18: E, n=4; M, n=9; L, n=5. Boxplot hinges indicate the 25th and 75th percentiles, whiskers extend from minimum to maximum values, all data points are shown, and horizontal bars denote the median. p-value calculated using a Mann-Whitney test comparing ALS4 and control donors within each age group.

**Extended Data Figure 9.**
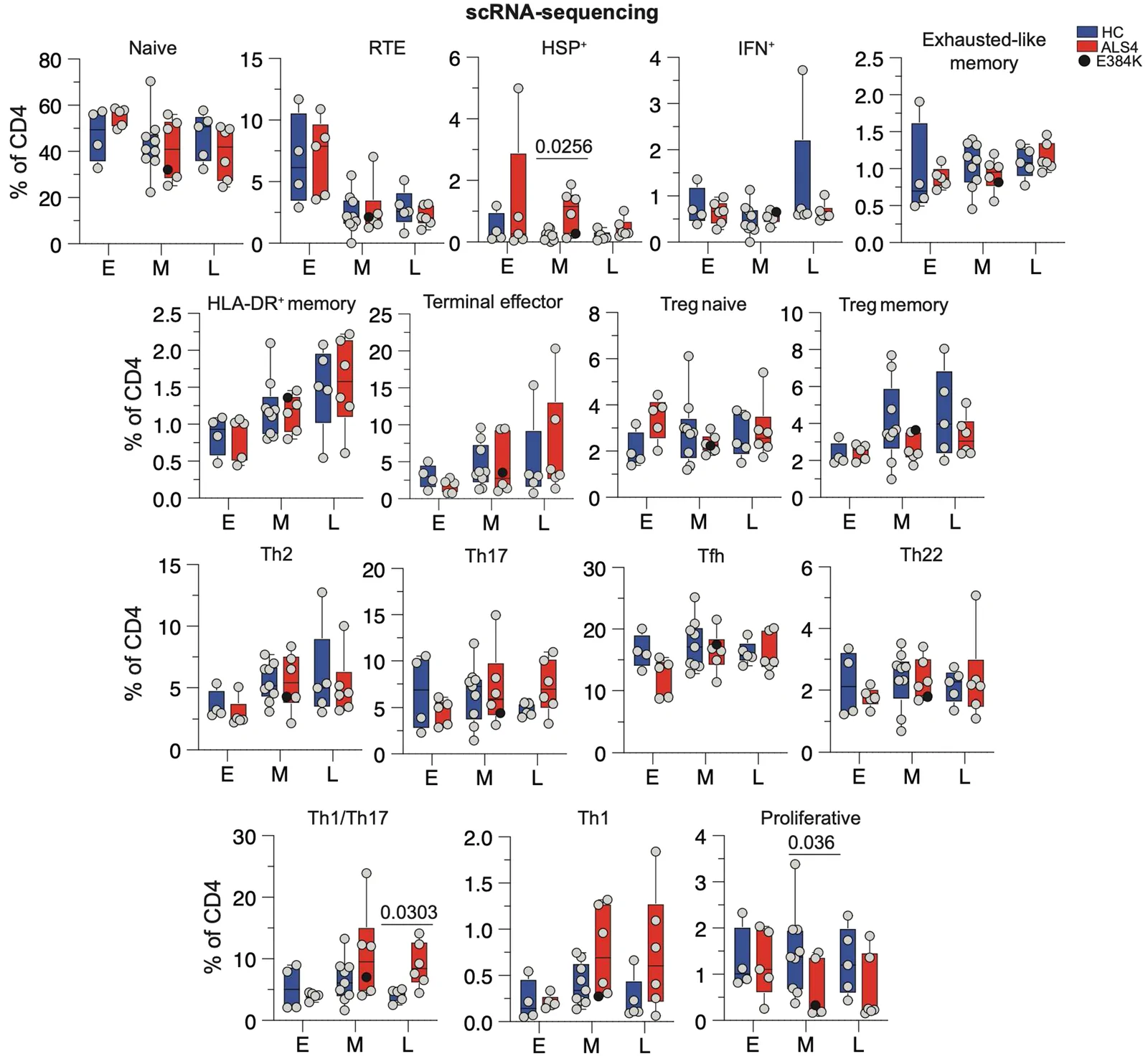
Related to Fig. 3b–d. Analysis of peripheral CD4 T cells by scRNA-seq. Peripheral CD4 T cells from HC and ALS4 patients were profiled by scRNA-seq. Boxplots of the relative distribution of the indicated subsets within the CD4 T cell compartment across HC and ALS4 groups. Black circle: ALS4 patient carrying the E384K variant. ALS4, n=17: E, n=5; M, n=6; L, n=6; HC, n=18: E, n=4; M, n=9; L, n=5. Boxplot hinges indicate the 25th and 75th percentiles, whiskers extend from minimum to maximum values, all data points are shown, and horizontal bars denote the median. p-value calculated using a Mann-Whitney test.

**Extended Data Figure 10.**
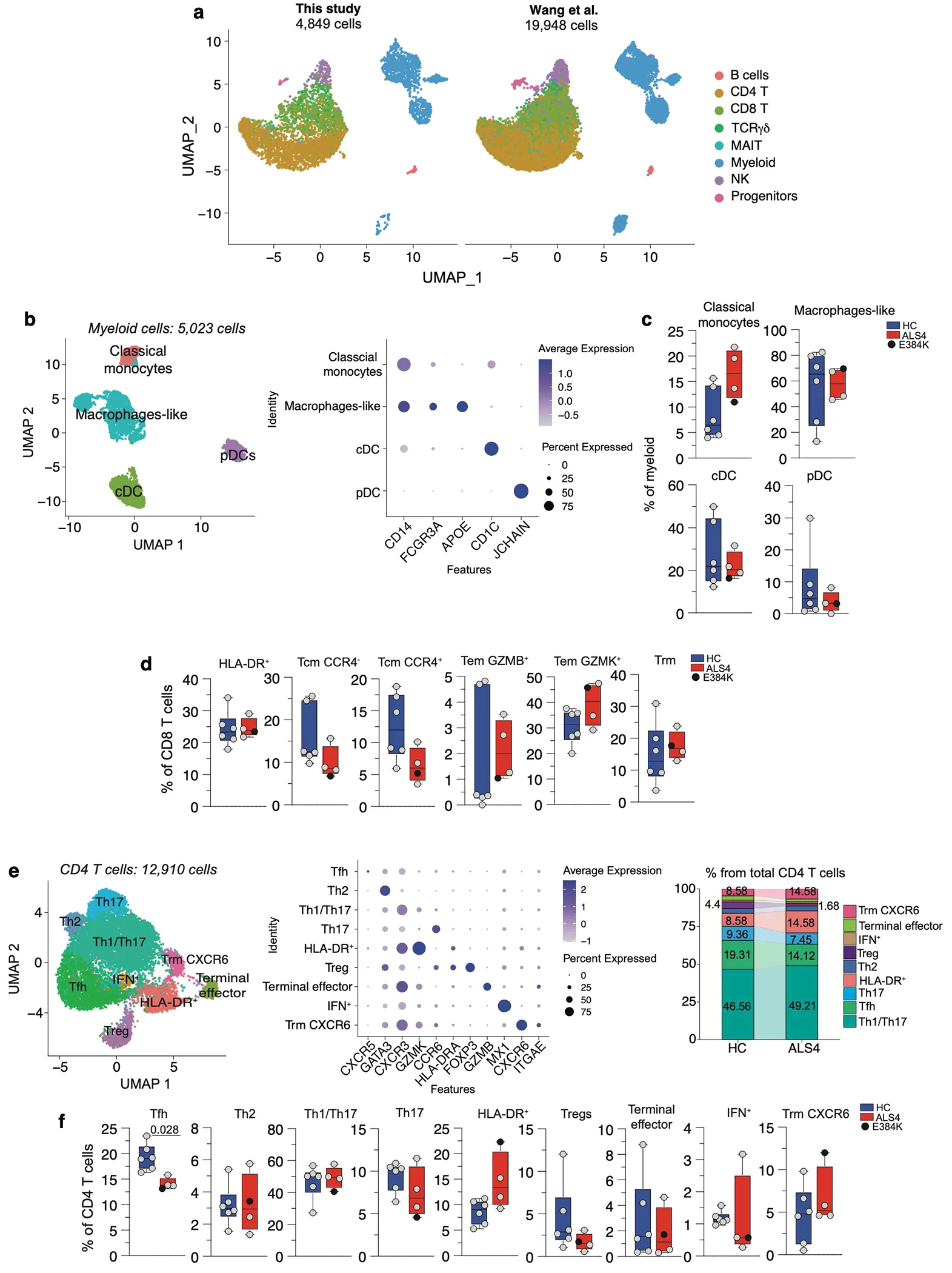
Related to Figure 4. CSF immune profiling in ALS4. CSF cells from ALS4 patients and HC were analyzed by scRNA-seq. **a.** UMAP of CSF cells from ALS4 patients and HC profiled in this study (**left**), together with CSF cells from five additional healthy controls reanalyzed from Wang et al., JCI Insight 2024 (**right**), with all cells colored by cluster identity. **b. Left**, UMAP projection of the indicate myeloid subsets colored by cluster identity. **Right**, dot plots showing expression of canonical marker genes used to delineate myeloid cell subsets. **c**. Boxplots of the relative distribution of the indicated subsets within the myeloid compartment. Black circle: ALS4 patient carrying the E384K variant. **d,f.** Boxplots of the relative distribution of the indicated subsets within CD8 (**d**) and CD4 (**f**) T cell compartments. Black circle: ALS4 patient carrying the E384K variant. **e. Left**, UMAP projection of the indicate CD4 T cell subsets colored by cluster identity. **Middle**, dot plots showing expression of canonical marker genes used to delineate CD4 T cell subsets. **Right**. Bar plots showing the relative distribution of CD8 T cell subsets across HC and ALS4 groups. ALS4, n=4; HC, n=6. Boxplot hinges indicate the 25th and 75th percentiles, whiskers extend from minimum to maximum values, all data points are shown, and horizontal bars denote the median. p-value calculated using a Mann-Whitney test.

**Extended Data Figure 11.**
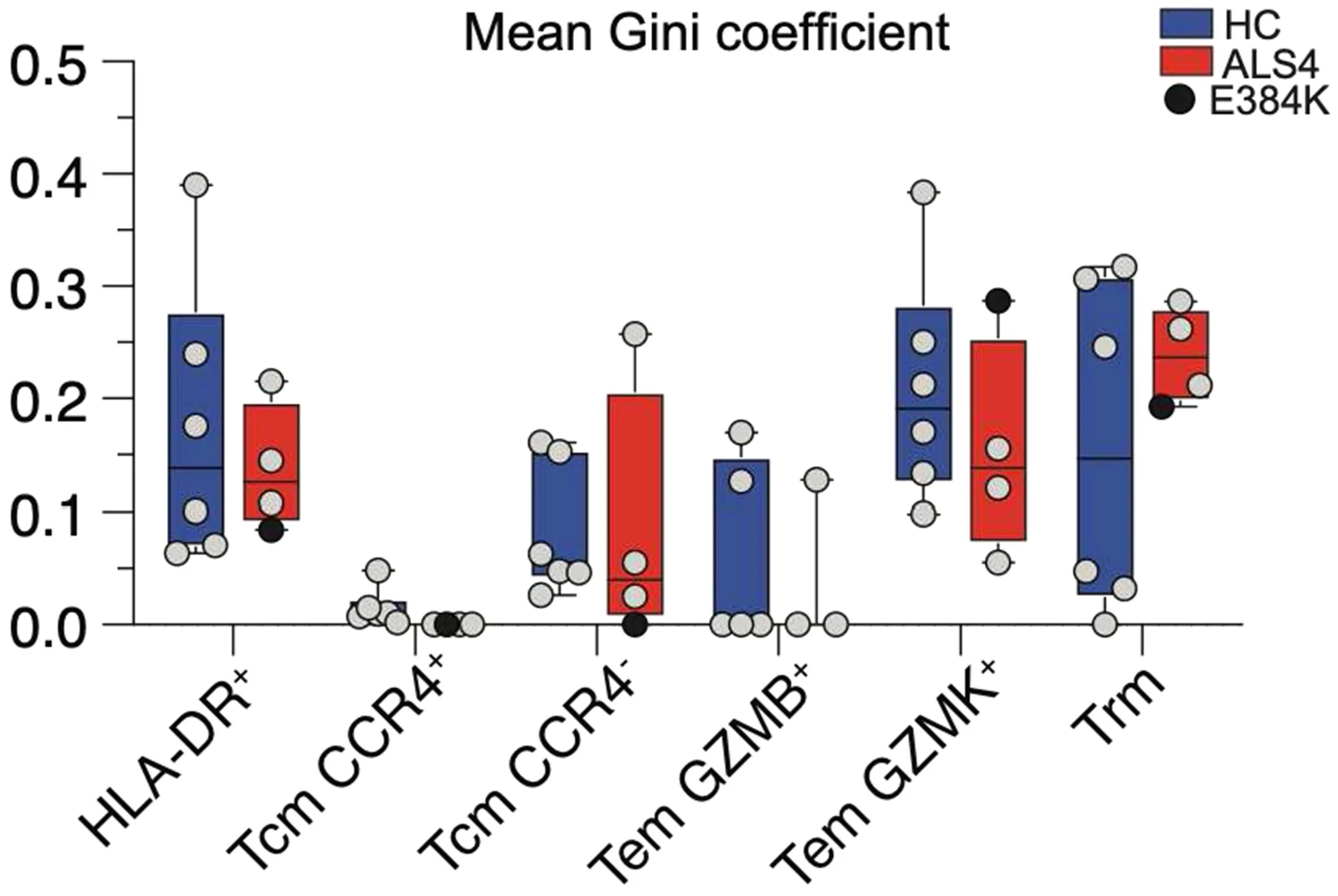
Related to Figure 4. Analysis of CD8 TCR repertoire in the CSF. CSF cells from ALS4 patients and HC were analyzed by scTCR-seq. Gini coefficients quantifying TCR repertoire inequality for the indicated CD8 T cell subsets in the CSF. Gini coefficients were calculated from TCR clonotype frequencies using 100 random subsamples per donor (Methods). Black circle: ALS4 patient carrying the E384K variant. ALS4, n=4; HC, n=6. Boxplot hinges indicate the 25th and 75th percentiles, whiskers extend from minimum to maximum values, all data points are shown, and horizontal bars denote the median.

## Supplementary Material

This is a list of supplementary files associated with this preprint. Click to download.

• SupplementaryTable3.pdf

• SupplementaryTable2.pdf

• SupplementaryTable5.pdf

• SupplementaryTable1.pdf

• SupplementaryTable4.pdf

## Figures and Tables

**Figure 1.**
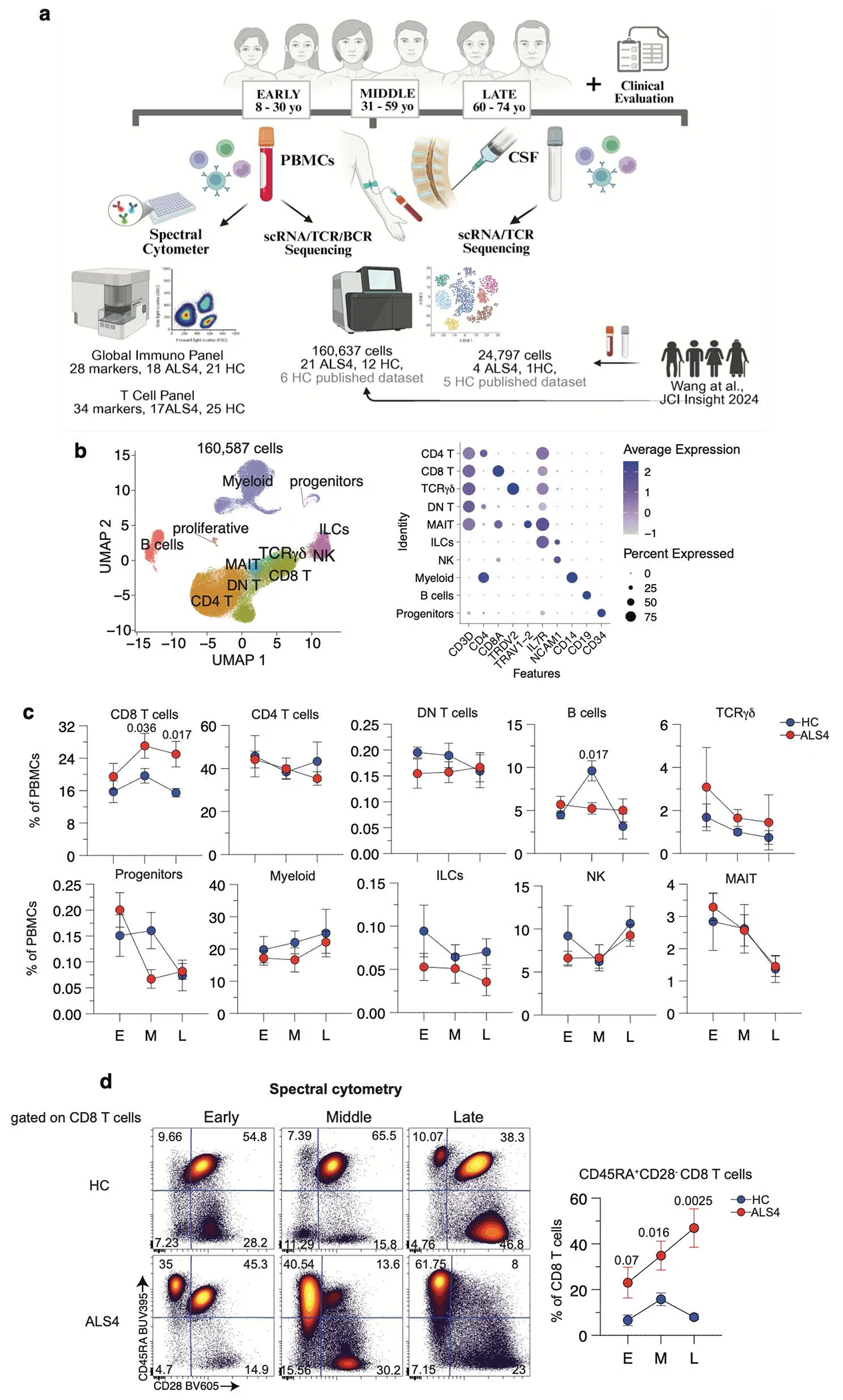
A CD8 T cell signature dominates the immune landscape in ALS4 across disease progression. **a.** Schematic of the study design. Peripheral blood mononuclear cells (PBMCs) and cerebrospinal fluid (CSF) from ALS4 patients spanning distinct disease stages and age-matched healthy controls (HC) were profiled by spectral flow cytometry and single-cell RNA/TCR/BCR sequencing. Donors were stratified into three age-defined groups: Early (E, 8-30 years-old, y.o.), Middle (M, 31-59 y.o.) and Late (L, 60-74 y.o.). **b. Left**, Uniform manifold approximation and projection (UMAP) of all PBMCs, colored by cell cluster identity. **Right**, dot plots depicting expression of canonical marker genes used to define immune subsets; color intensity reflects the mean expression level within each cluster, and dot size indicates the proportion of cells in that cluster expressing the marker. **c.** Relative frequency of each PBMC cluster in ALS4 patients and HC across the three age groups. ALS4, n=17: E, n=5; M, n=6; L, n=6; healthy controls, n=18: E, n=4; M, n=9; L, n=5. mean ± SEM, p-value calculated using a Mann-Whitney test**. d**. CD8 T cells in PBMCs were profiled by spectral flow cytometry after gating on CD14^−^ CD19^−^ TCRγδ^−^ TCR Vα7.2^−^ CD56^−^ CD3^+^ CD8^+^ cells. **Left**, representative dot plots show concatenated data from donors in each age group. **Right**, frequencies of CD45RA^high/+^ CD28^−^ CD8 T cells are shown for ALS4 patients (n=17: E, n=3; M, n=7; L, n=7) and HC (n=25: E, n=5; M, n=15; L, n=5); mean ± SEM, p-values calculated using a Mann-Whitney test comparing ALS4 and control donors within each age group.

**Figure 2.**
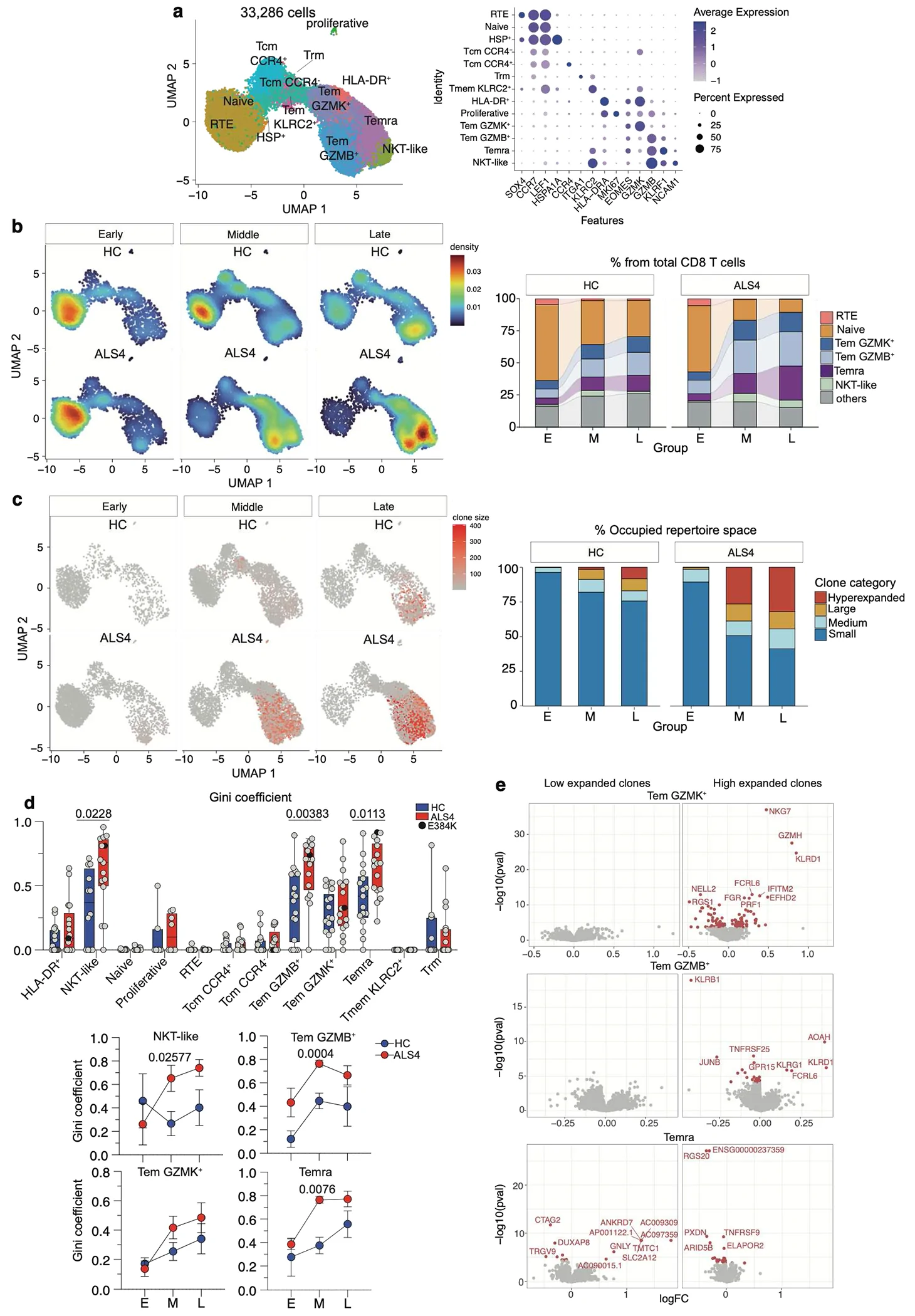
Clonally expanded GZMB^+^ CD8 T cells define ALS4 from early disease stages. Peripheral CD8 T cells from HC and ALS4 patients were analyzed by scRNA/TCR-seq. **a. Left**, UMAP projection of CD8 T cells colored by cluster identity. **Right**, dot plots showing expression of canonical marker genes used to delineate CD8 T cell subsets. **b.** UMAP density plots (**left**) and corresponding bar plots (**right**) of the relative distribution of CD8 T cell subsets across HC and ALS4 groups and age strata. **c. Left**, Single-cell TCRαβ analysis overlaid on the CD8 T cell UMAP, highlighting clonal size distribution. **Right**, proportions of clonally expanded CD8 T cells classified as hyperexpanded (>100 cells), large (20 < x ≤ 100 cells), medium (5 < x ≤ 20 cells) and small (≤5 cells). **d.** Gini coefficients quantifying TCR repertoire inequality for the CD8 T cell subsets (top), and for selected CD8 T cell subsets across age groups (bottom). Black circle: ALS4 patient carrying the E384K variant. mean ± SEM, p-value calculated using a Mann-Whitney test comparing ALS4 and control donors within each age group. Boxplot hinges indicate the 25th and 75th percentiles, whiskers extend from minimum to maximum values, all data points are shown, and horizontal bars denote the median. **e**. Differential gene expression (DE) analysis of low-versus high-expanded clones within the indicated CD8 T cell subsets in ALS4 patients. Volcano plots display genes significantly up- or downregulated when comparing low-expanded clones (≤5 cells per clonotype) to high-expanded clones (>20 cells per clonotype) within the same CD8 T cell subset (See Methods). ALS4, n=17: E, n=5; M, n=6; L, n=6; HC, n=18: E, n=4; M, n=9; L, n=5.

**Figure 3.**
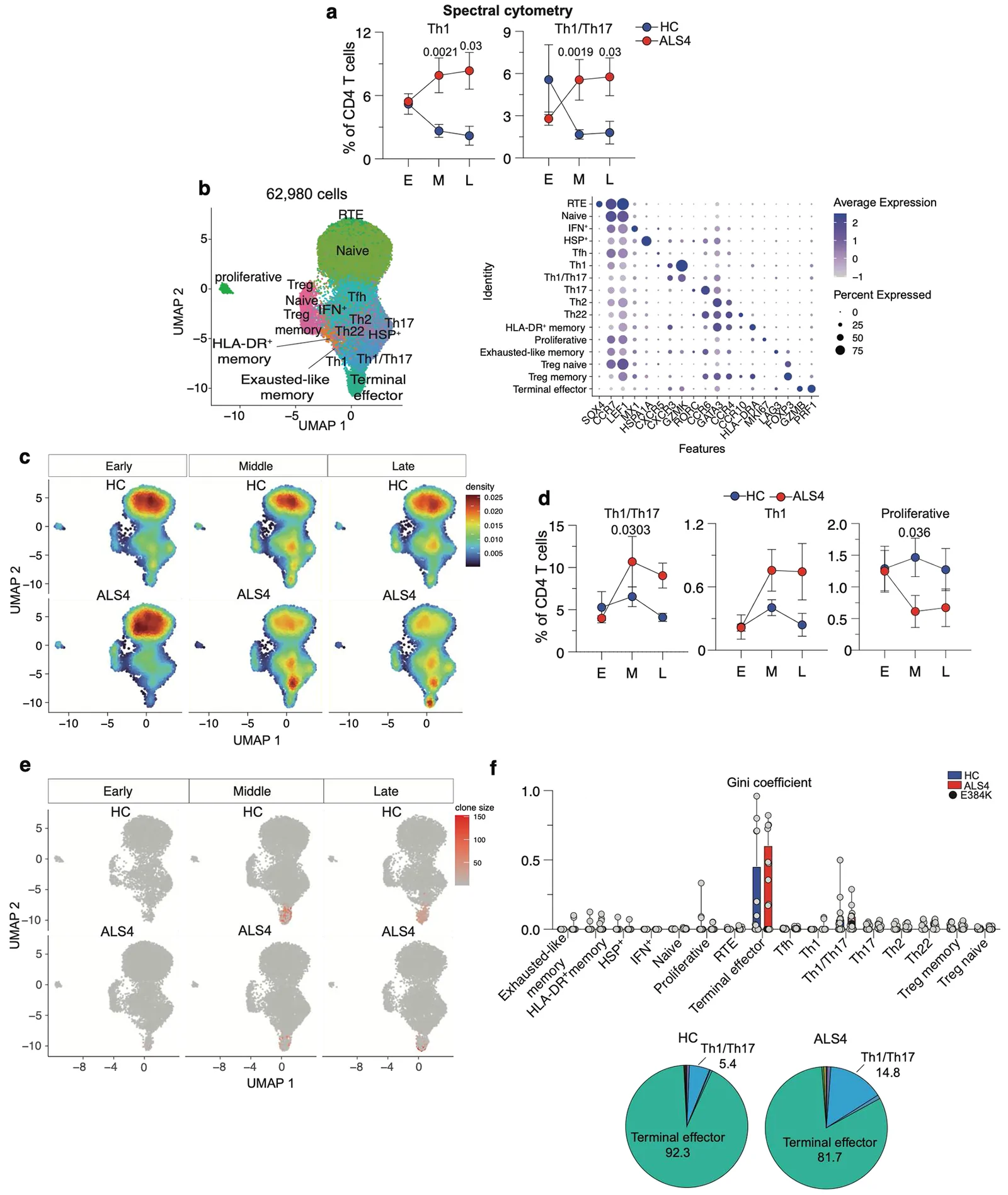
Altered frequencies of peripheral Th1 and Th1/Th17 CD4 T cells during ALS4 progression. Peripheral CD4 T cells from HC and ALS4 patients were profiled by spectral flow cytometry (**a**) and scRNA/TCR sequencing (**b-f**). **a.** Frequencies of Th1 and Th1/Th17 within CD4 T cells in the PBMCs of HC and ALS4 patients determined by spectral cytometry analysis. n=17 ALS4: E, n=3; M, n=7; L, n=7; n=25 HC: E, n=5; M, n=15; L, n=5. mean ± SEM, p-values calculated using a Mann-Whitney test comparing ALS4 and control donors within each age group. **b. Left**, UMAP projection of CD4 T cells colored by cluster identity. **Right**, dot plots showing expression of canonical marker genes used to delineate CD4 T cell subsets. **c.** UMAP density plots of the relative distribution of CD4 T cell subsets across HC and ALS4 groups and age strata. **d.** Relative frequencies of the indicated CD4 T cell subsets in ALS4 patients and HC. **e.** Single cell TCRαβ analysis overlaid on the CD4 T cell UMAP, highlighting clonal size distribution. **f. Top.** Gini coefficients quantifying TCR repertoire inequality for the CD4 T cell subsets. Black circle: ALS4 patient carrying the E384K variant**. Bottom**, Pie charts showing the frequency of shared terminal effector TCRαβ clonotypes with the same or different blood cell types, stratified by disease. ALS4, n=17: E, n=5; M, n=6; L, n=6; HC, n=18: E, n=4; M, n=9; L, n=5. mean ± SEM, p-values calculated using a Mann-Whitney test comparing ALS4 and control donors within each age group. Boxplot hinges indicate the 25th and 75th percentiles, whiskers extend from minimum to maximum values, all data points are shown, and horizontal bars denote the median.

**Figure 4.**
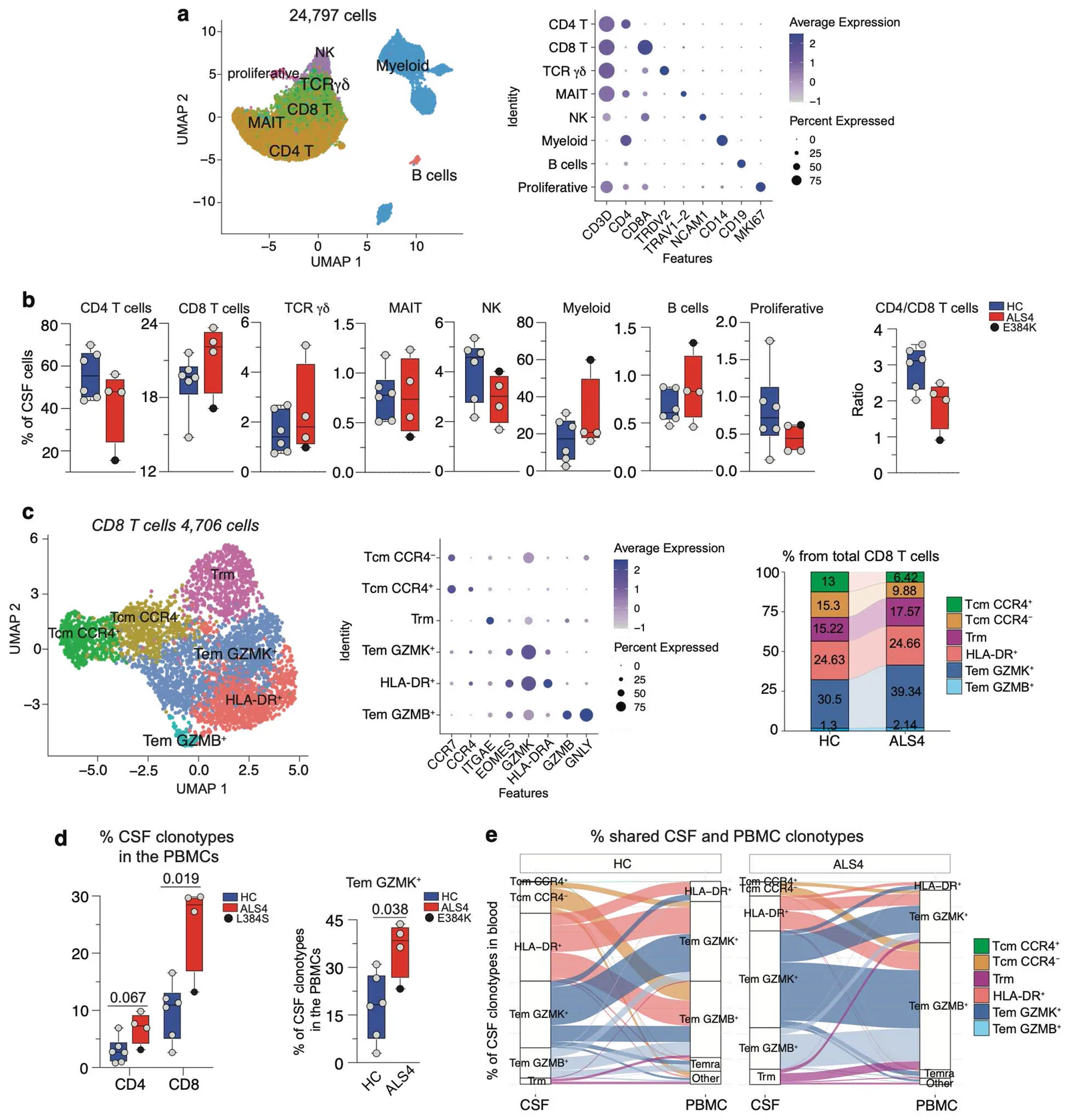
CSF immune profiling reveals shared GZMK-GZMB CD8 T cell clonotypes between CSF and blood in ALS4 patients. CSF from ALS4 patients and HC were profiled by scRNA/TCR sequencing. **a. Left**, UMAP projection of total CSF cells colored by cluster identity. **Right**, dot plots showing expression of canonical marker genes used to delineate CSF cell subsets. **b.** Relative frequencies of the indicated CSF cell subsets and CD4/CD8 T cell ratios in HC and ALS4 patients. Black circle: ALS4 patient carrying the E384K variant. **c. Left**, UMAP projection of CD8 T cells colored by cluster identity. **Middle**, Dot plots showing expression of canonical marker genes used to delineate CD8 T cell subsets. **Right**, Bar plots showing the relative distribution of CD8 T cell subsets across HC and ALS4 groups. **d.** Frequencies of unique CSF CD4 and CD8 T cell clonotypes that are shared with PBMCs in HC and ALS4 patients (**left**) and the corresponding frequencies for the CD8 Tem GZMK^+^ subset specifically (**right**). Black circle: ALS4 patient carrying the E384K variant **e.** Relative distribution of subset phenotypes between shared CSF-PBMC clonotypes. ALS4, n=4; HC, n=6. In **b** and **d**, boxplot hinges indicate the 25th and 75th percentiles, whiskers extend from minimum to maximum values, all data points are shown, and horizontal bars denote the median. p-value calculated using a Mann-Whitney test.

## Data Availability

All single-cell datasets and code required to reproduce the figures will be publicly available upon publication.
